# The Double Burden of Malnutrition in the Brazilian Legal Amazon: Spatial Distributions and Temporal Trends (2013–2023)

**DOI:** 10.3390/nu17061054

**Published:** 2025-03-17

**Authors:** Alanderson Alves Ramalho, Tamires Mota da Silva, Yara de Moura Magalhães Lima, Tiago Feitosa da Silva, Michelle Adler de Oliveira, Suellen Cristina Enes Valentim da Silva, Maria Eduarda Alves Anute, Eduardo Batista Barbosa, Danila Torres de Araujo Frade Nogueira, Flávia Santos Batista Dias

**Affiliations:** 1 Postgraduate Program in Public Health, Federal University of Acre, Rio Branco 69920-900, AC, Brazil; tamires.mota@ufac.br (T.M.d.S.); yara.moura@sou.ufac.br (Y.d.M.M.L.); tiago.feitosa@sou.ufac.br (T.F.d.S.); michelle.oliveira@sou.ufac.br (M.A.d.O.); suellen.silva@ifac.edu.br (S.C.E.V.d.S.); 2 Center for Health Sciences and Sports, Federal University of Acre, Rio Branco 69920-900, AC, Brazil; danila.nogueira@ufac.br (D.T.d.A.F.N.); flavia.dias@ufac.br (F.S.B.D.); 3 Center for Health Sciences and Sports, Bachelor’s Degree in Nutrition, Federal University of Acre, Rio Branco 69920-900, AC, Brazil; maria.anute@sou.ufac.br (M.E.A.A.); eduardo.batista@sou.ufac.br (E.B.B.)

**Keywords:** nutritional assessment, undernutrition, obesity, time series

## Abstract

Malnutrition and overweight are significant public health challenges, especially in low- and middle-income countries. In the Amazon, high rates of malnutrition and the alarming rise of obesity highlight persistent regional inequalities. Therefore, the aim of this study was to analyze spatial distributions and temporal trends of malnutrition among children under five, adults, and elderly individuals in primary healthcare services across municipalities in the Legal Amazon from 2013 to 2023. Methods: This ecological study used data from the Food and Nutritional Surveillance System (SISVAN). The analysis included the prevalences of underweight, overweight, and obesity, stratified by year, sex, and federative unit. Spatial distributions were analyzed using QGIS version 3.22, and temporal trends were assessed with Joinpoint version 4.6 and expressed as annual percentage changes (APCs) and 95% confidence intervals (95% CIs). Results: Between 2013 and 2023, SISVAN monitored 10,451,758 children under five years, 30,831,720 adults, and 4,456,650 elderly individuals in the Legal Amazon. Stunting in children under five years decreased from 20.45% to 15.30%, with a significant downward trend (APC: −2.7; 95% CI: −3.7; −1.6). Childhood overweight exhibited a general downward trend but with notable fluctuations. Overweight in adults increased from 48.85% to 64.64%, and obesity from 17.10% to 28.49%, both showing significant upward trends. For the elderly, underweight decreased from 17.47% to 13.04%, with a downward trend until 2018, while overweight increased from 42.35% to 48.22%, with a significant upward trend until 2017. Conclusions: Despite progress in reducing childhood underweight, its prevalence in the Legal Amazon remains high compared to national averages. Significant regional disparities and rising overweight rates among adults and elderly individuals indicate a need for targeted public health strategies to address these nutritional issues.

## 1. Introduction

Malnutrition represents a global challenge that profoundly impacts the health and wellbeing of populations worldwide [1]. This issue, which includes undernutrition alongside overweight, obesity, and diet-related diseases, predominantly affects low- and middle-income countries [2]. Undernutrition can manifest as insufficient bodyweight and a deficiency of essential nutrients, whereas overweight and obesity result from excessive fat accumulation, often linked to excessive energy intake, primarily from ultra-processed foods, a poor diet, and other unhealthy lifestyle habits [3]. Approximately 45% of the deaths among children under five are associated with undernutrition, which predominantly occurs in low- and middle-income countries. At the same time, these countries are witnessing rising prevalences of childhood overweight and obesity [1].

In 2020, it was estimated that 149 million children under the age of five experienced stunting, characterized by a low height-for-age value, which is an indicator of chronic malnutrition; 45 million experienced wasting, indicated by a low weight-for-height value, a sign of acute malnutrition; and approximately 39 million were overweight or obese [1]. Estimates suggest that in adults, approximately 1.9 billion are overweight or obese, while 462 million are underweight [1].

In recent years, Brazil has undergone an important nutritional transition, marked by a reduction in undernutrition and a concerning rise in obesity among young people and adults. Although undernutrition has decreased over the decades, it still affects specific segments of the population. For instance, the prevalence of undernutrition among black boys reduced from 5.2% in 2015 to 4.8% in 2018, but it increased again to 5.6% in 2019 and 5.3% in 2021 [4].

Meanwhile, the rise in obesity presents an even more alarming scenario. According to the 2019 National Health Survey (PNS), the proportion of obese adults in Brazil more than doubled between 2003 and 2019, rising from 12.2% to 26.8%. Among women, obesity increased from 14.5% to 30.2%, while among men, it rose from 9.6% to 22.8% [5]. Furthermore, overweight and obesity have significantly contributed to the increase in deaths associated with these conditions. Between 1990 and 2019, deaths from overweight and obesity in Brazil rose from 74,266 to 177,940 cases [6].

In Latin America and the Caribbean, malnutrition presents significant diet-related public health challenges. According to Popkin and Reardon (2018), this nutritional transition is driven by structural changes in food systems, characterized by the expansion of supermarket chains, increased availability of ultra-processed foods, and the adoption of Western dietary patterns. These transformations are linked to long-term alterations in employment patterns, urbanization, and demographic dynamics, which modify eating habits and physical activity practices [2]. Undernutrition and excess bodyweight are prevalent issues in Latin American countries. Overweight and obesity often coexist with undernutrition at the national level, indicating a common occurrence of the double burden. This co-occurrence highlights the presence of the double burden in Latin American countries [7].

Countries such as Brazil have experienced a significant shift in the nutritional profile of their populations. In Brazilian state capitals, the double burden of malnutrition among adults is more pronounced among older women with low levels of education, whereas overweight is more prevalent among men with higher levels of education [8]. Between 1975 and 2019, underweight among men decreased from 9.1% to 2.5% and among women from 12.2% to 3.4%. Conversely, obesity rates increased from 3% to 22% among men and from 9% to 30% among women, showing a direct and inverse relationship with income quintiles [9].

In the Legal Amazon, a vast region encompassing nine Brazilian states and characterized by extensive ecological and cultural diversities, malnutrition exhibits distinct characteristics [10]. This region faces both high rates of undernutrition and increasing prevalences of overweight and obesity. The Global Burden of Disease study (1990–2019) found that the north region of Brazil, which includes seven states from the Legal Amazon, and the northeast had the highest mortality rates because of malnutrition in the country. A downward trend in mortality related to high BMIs was also observed in the wealthier regions (south, southeast, and center west), while these rates increased in the regions with the lowest sociodemographic index (northeast and north) [11]. The complexity of these issues is exacerbated by socioeconomic inequalities and difficulties in accessing nutritious food and health services [12].

The developmental, economic, social, and health impacts of the global burden of malnutrition, in all its forms, are severe and long lasting, affecting individuals and their families, communities, and countries [13]. Therefore, investigating the nutritional situation in remote regions, such as the Brazilian Amazon, is essential for a better understanding of how these conditions interact and evolve over time.

Despite some improvements in reducing underweight at the national level, the Amazon region continues to exhibit high rates of malnutrition, reflecting significant disparities compared to national averages [11]. Furthermore, the concerning increases in the prevalences of overweight and obesity among adults and the elderly at the national level [8,14] also appear to be occurring in the Amazon region [11].

Given the importance of monitoring these indicators in this region, this study aimed to analyze the spatial distributions and temporal trends of undernutrition and overweight in children under five, adults, and the elderly receiving care at primary healthcare facilities in municipalities within the Legal Amazon from 2013 to 2023. By highlighting regional disparities and trends over time, we aim to contribute to the development of strategies to improve nutrition and health in the Legal Amazon.

## 2. Materials and Methods

This ecological study utilized data from the Food and Nutrition Surveillance System (SISVAN) to analyze the temporal trends of malnutrition in children under five years of age, adults, and the elderly receiving care at primary healthcare facilities in the municipalities of the Legal Amazon of Rio Branco, Acre, from 2013 to 2023. The primary objective of SISVAN is to provide continuous information on nutritional and dietary status with a focus on vulnerable groups. However, as a limitation, it is restricted to the population served by primary healthcare within the Unified Health System.

**Figure 1:**
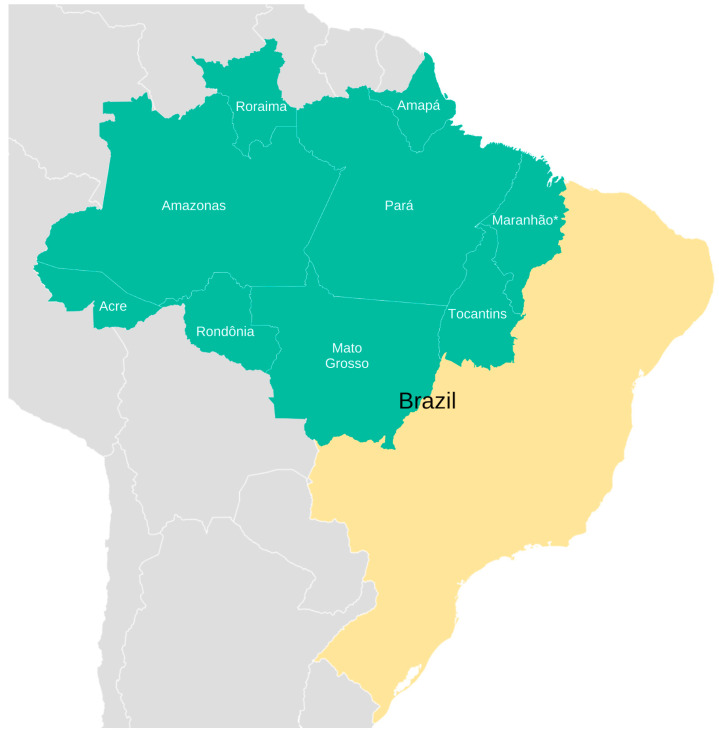
Map showing the Brazilian states that make up the Legal Amazon (in green) and their location in Brazil (in yellow).

The Brazilian Legal Amazon encompasses an area of 5,217,423 km^2^, representing 61% of the Brazilian territory (Figure 1). This region includes the entire Brazilian Amazon biome, 20% of the Cerrado biome, and a portion of the Pantanal biome in Mato Grosso. It covers all the states of Acre, Amapá, Amazonas, Mato Grosso, Pará, Rondônia, Roraima, and Tocantins, as well as a part of Maranhão, totaling 772 municipalities [15]. The study population comprises all the children under five years of age, adults, and the elderly who received care at primary healthcare facilities within the 772 municipalities of the Legal Amazon between 2013 and 2023 and had their anthropometric assessments recorded in SISVAN.

The study variables include the prevalences of undernutrition, overweight, and obesity in children under five years of age, adults, and the elderly, stratified by reference year, sex, and federative unit.

To estimate the nutritional status of the children under five, the anthropometric indices weight for height (W/H) and height for age (H/A) were utilized [16]. The prevalence of acute malnutrition was determined based on wasting (characterized by a low weight-for-height value in the W/H index, with a Z-score of <−2), and the prevalence of chronic malnutrition was determined based on stunting (characterized by a low height-for-age value in the H/A index, with a Z-score of <−2). Overweight prevalence was assessed using the W/H index, with a Z-score of >+2 indicating overweight and a Z-score of >+3 indicating obesity. The nutritional statuses of the adults and elderly were estimated using the body mass index (BMI). In adults, underweight prevalence was determined based on underweight (a BMI of <18.5 kg/m^2^), overweight prevalence was determined based on a BMI of ≥25.0 kg/m^2^, and obesity was defined based on a BMI of ≥30.0 kg/m^2^ [17]. In the elderly, underweight was defined based on a BMI of <22.0 kg/m^2^, and overweight was defined based on a BMI of ≥27.0 kg/m^2^ [18]. Interobserver variability assessments were not conducted. Measurements were performed by experienced healthcare professionals in anthropometry, following the WHO recommendations for anthropometric assessments [19].

Data analysis was initially performed descriptively, with absolute and relative frequencies of the variables presented by reference year.

For the spatial distribution analysis, geographic processing and mapping were conducted using QGIS software, version 3.22, applying the planar coordinate system, Universal Transverse Mercator System (UTM), and SIRGAS 2000 data obtained from the European Petroleum Survey Group (EPSG): 4674. To facilitate visualization, choropleth maps, illustrating the prevalence of each outcome, were generated. Temporal trend analyses were conducted using Joinpoint software, version 4.6.00, which identifies changes in indicator trends over time through inflection points. For model adjustment, 0 to 3 joinpoints were allowed, and annual percentage changes (APCs) were estimated with 95% confidence intervals (95% CIs). The number of significant joinpoints was determined by performing multiple permutation tests (default: 4499 permutations). The tests’ performance was assessed using Monte Carlo simulations, considering potential autocorrelated errors [20]. The results were expressed as annual percentage changes (APCs) with their respective 95% CIs.

The data used in this research are publicly available and accessible, provided by the Ministry of Health in an unrestricted manner and without any personal identification. Therefore, this research does not require informed consent or ethical review under CNS Resolution 466/12.

## 3. Results

From 2013 to 2023, the SISVAN monitored 10,451,758 children under five years of age, 30,831,720 adults, and 4,456,650 elderly individuals residing in the Brazilian Legal Amazon.

### 3.1. Children Under Five Years of Age

The prevalence of chronic malnutrition in children under five years, estimated based on stunting, decreased from 20.45% in 2013 to 15.30% in 2023, showing a significant downward trend throughout the period (APC: −2.7; 95% CI: −3.7, −1.6, Table 1) in the Legal Amazon. When stratified by federative unit, the same downward trend was observed, with variations across different periods (Table S1). It was observed that only the states of Rondônia and Mato Grosso had prevalences below the national prevalence of 11.8% in 2023 (Table S1).

**Figure 2:**
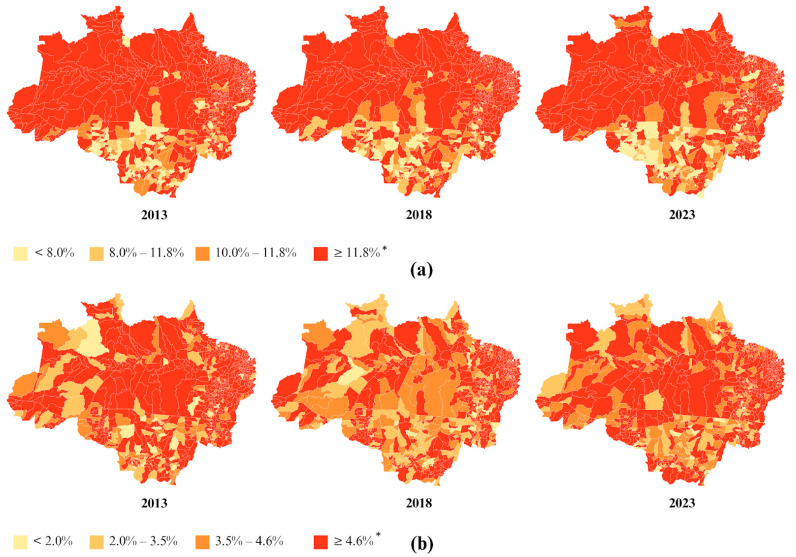
Prevalences of stunting (**a**) and wasting (**b**) in children under 5 years old, stratified by municipality, in the Legal Amazon in 2013, 2018, and 2023. * National prevalence in 2023.

The spatial distribution of chronic malnutrition in children under five years, stratified by municipality, in the Legal Amazon in 2023 is presented in Figure 2. When stratified by municipality, 532 municipalities (69% of the total) had prevalences above the national average.

The prevalence of acute malnutrition in children under five years, estimated based on wasting, varied from 6.86% in 2013 to 5.84% in 2023, with no significant change observed throughout the period (APC: −0.5; 95% CI: −1.9, 0.9) in the Legal Amazon (Table 1). When stratified by state, only Acre (APC: −4.1; 95% CI: −11.1, −1.6; period: 2013–2018; APC: 2.4; 95% CI: −0.5, 9.6; period: 2018–2023) and Roraima (APC: −15.9; 95% CI: −20.5, −12.9; period: 2013–2023) exhibited significant downward trends (Table S2).

The spatial distribution of acute malnutrition in children under five years, stratified by state, in the Legal Amazon in 2023 showed that only the state of Amapá (4.13%) had a prevalence below the national prevalence of 4.55% in 2023 (Table S2). When stratified by municipality, 517 municipalities (67% of the total) had prevalences above the national average (Figure 2).

The prevalence of overweight in children under five years varied from 15.23% in 2013 to 11.80% in 2023, showing a downward trend during the period from 2013 to 2018 (APC: −4.6; 95% CI: −6.5, −3.7), an upward trend during 2018–2021 (APC: 3.9; 95% CI: 1.2, 5.8), and a downward trend from 2021 to 2023 (APC: −9.4; 95% CI: −13.6, −5.1) in the Legal Amazon (Table 1).

**Figure 3:**
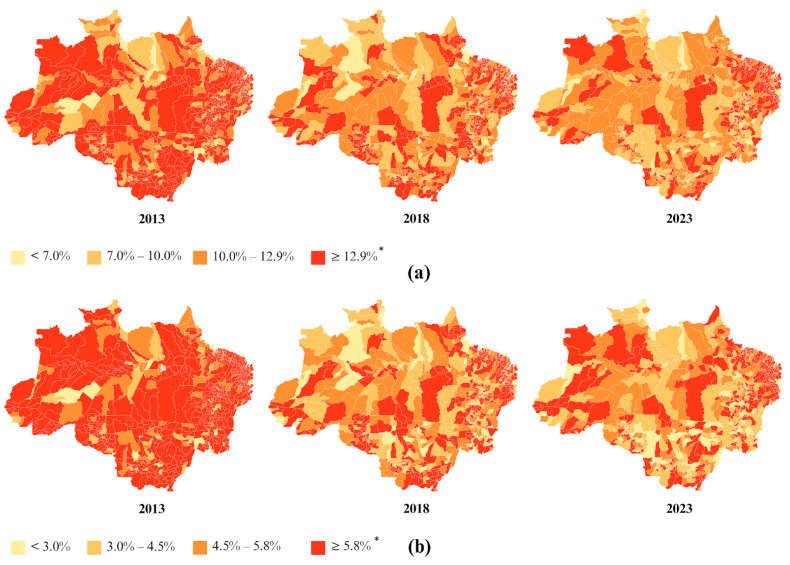
Prevalences of overweight (**a**) and obesity (**b**) in children under 5 years old, stratified by municipality, in the Legal Amazon in 2013, 2018, and 2023. * National prevalence in 2023.

The spatial distribution of overweight in children under five years, stratified by state, in the Legal Amazon in 2023 indicated that only the state of Maranhão (14.24%) had a prevalence above the national prevalence of 13.47% in 2023 (Table S3). When stratified by municipality, 276 municipalities (48% of the total) had prevalences above the national average (Figure 3).

The prevalence of obesity in children under five years varied from 9.04% in 2013 to 5.42% in 2023, showing a downward trend from 2013 to 2018 (APC: −9.0; 95% CI: −11.6, −7.7), an upward trend from 2018 to 2021 (APC: 5.5; 95% CI: 1.2, 8.9), and a downward trend from 2021 to 2023 (APC: −13.4; 95% CI: −19.2, −7.0) in the Legal Amazon (Table 1).

The spatial distribution of obesity in children under five years, stratified by state, in the Legal Amazon in 2023 revealed that the states of Acre (6.12%) and Maranhão (7.10%) had prevalences above the national prevalence of 5.84% in 2023 (Table S4). When stratified by municipality, 322 municipalities (41.71% of the total) had prevalences above the national average (Figure 3).

### 3.2. Adults

The prevalence of underweight in adults decreased from 3.87% in 2013 to 2.26% in 2023, showing a downward trend during the periods 2013–2015 (APC: −13.5; 95% CI: −15.8, −9.4) and 2015–2021 (APC: −5.2; 95% CI: −6.7, −3.9) and an insignificant variation during 2021–2023 in the Legal Amazon (Table 2).

**Figure 4:**
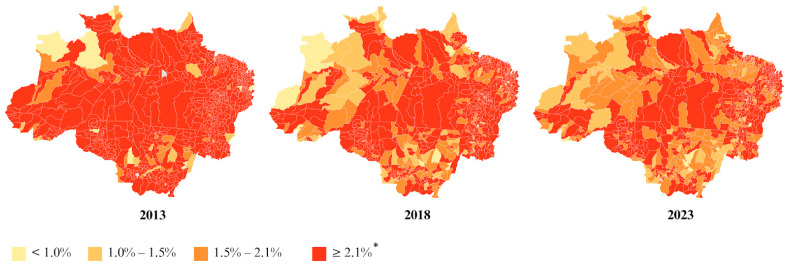
Prevalence of underweight in adults, stratified by municipality, in the Legal Amazon in 2013, 2018, and 2023. * National prevalence in 2023.

The spatial distribution of underweight in adults, stratified by state, in the Legal Amazon in 2023 indicated that the states of Acre (2.13%), Rondônia (2.56%), Tocantins (2.33%), and Maranhão (2.64%) had prevalences above the national prevalence of 2.10% in 2023 (Table S5). When stratified by municipality, 319 municipalities (41.32% of the total) had prevalences above the national average (Figure 4).

The prevalence of overweight in adults increased from 48.85% in 2013 to 64.64% in 2023 in the Legal Amazon. During the same period, the prevalence of obesity increased from 17.10% to 28.49%, showing a significant upward trend (Table 2). When stratified by federative unit, the same upward trend was observed, with variations across different periods. Tables S6 and S7 detail the annual percentage changes, stratified by sex and federative unit.

**Figure 5:**
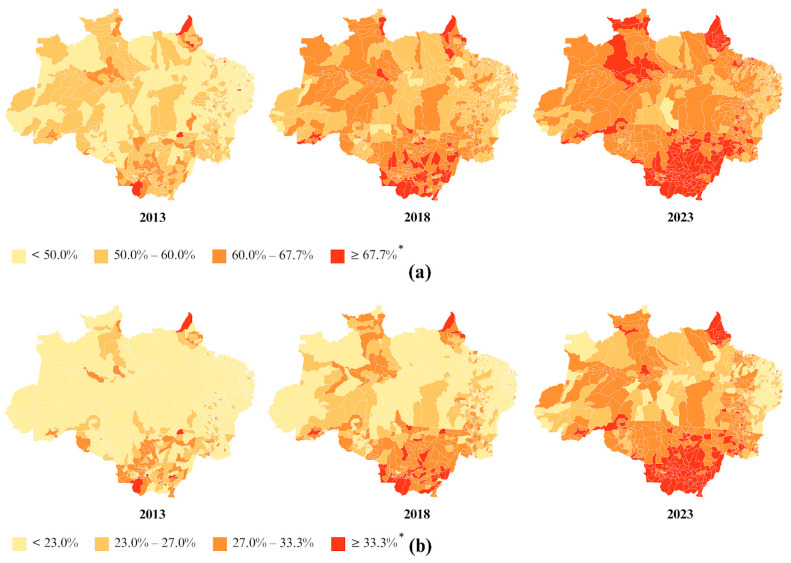
Prevalences of overweight (**a**) and obesity (**b**) in adults, stratified by municipality, in the Legal Amazon in 2013, 2018, and 2023. * National prevalence in 2023.

In the spatial distribution analysis, it was observed that in 2023, 183 Amazonian municipalities (24% of the total) had prevalences above the national prevalence of 67.7% for overweight in adults, and 128 Amazonian municipalities (17% of the total) had prevalences above the national prevalence of 33.3% for obesity in adults (Figure 5).

### 3.3. Elderly

The prevalence of underweight in the elderly decreased from 17.47% in 2013 to 13.04% in 2023, showing a downward trend from 2013 to 2018 (APC: −6.3; 95% CI: −8.3, −5.2) and an insignificant variation from 2018 to 2023 in the Legal Amazon (Table 3).

**Figure 6:**
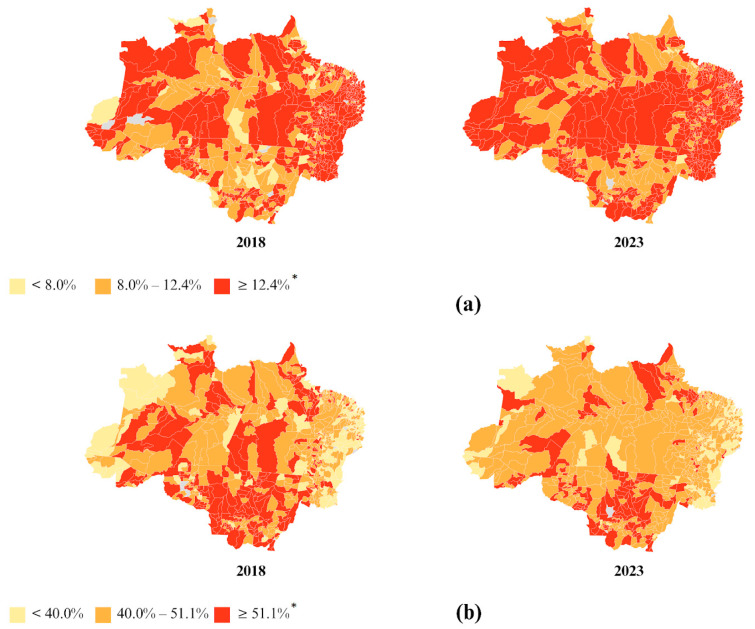
Prevalences of underweight (**a**) and overweight (**b**) in older adults, stratified by municipality, in the Legal Amazon in 2018 and 2023. * National prevalence in 2023.

The spatial distribution of underweight in the elderly, stratified by state, in the Legal Amazon in 2023 indicated that the states of Acre (14.72%), Pará (12.96%), Rondônia (12.50%), Tocantins (14.89%), and Maranhão (14.85%) had prevalences above the national prevalence of 12.36% in 2023 (Table S8). When stratified by municipality, 212 municipalities (27.46% of the total) had prevalences above the national average (Figure 6).

The prevalence of overweight in the elderly increased from 42.35% in 2013 to 48.22% in 2023, showing a significant upward trend from 2013 to 2017 (APC: 3.4; 95% CI: 2.5, 5.1) and no significant variation from 2017 to 2023 (APC: 0.1; 95% CI: −0.7, 0.6) in the Legal Amazon (Table 3).

The spatial distribution analysis indicated that only the states of Amazonas (51.74%) and Mato Grosso (52.46%) had prevalences above the national prevalence of 51.12% in 2023 (Table S9). When stratified by municipality, 145 municipalities (19% of the total) had prevalences above the national average (Figure 6).

## 4. Discussion

This study analyzed the evolutions of undernutrition and overweight prevalences among children under five, adults, and the elderly in the Legal Amazon over a decade (2013–2023). The results reveal a significant reduction in the prevalence of chronic malnutrition among children and a complex variation in childhood overweight. In contrast, there were consistent increases in the prevalences of overweight and obesity among adults, along with a significant rise in overweight among the elderly.

Similarly, many countries have experienced the double burden of malnutrition, characterized by the coexistence of overweight and undernutrition within the same population, or even in the same individual. However, the downward trend in underweight has led to its underestimation as a public health priority compared to the focus on overweight and obesity. This makes understanding and controlling malnutrition, in all its forms, a public health challenge [21,22,23,24].

The results for the Legal Amazon, regarding the double burden of malnutrition, reveal significant regional disparities when compared to national averages. Despite the reduction in the prevalence of chronic malnutrition in children under five, it still remains above the national average [25]. These disparities can be attributed to factors such as limited access to health services and inadequate infrastructure in more remote regions of the Amazon [26,27].

The reduction in the prevalence of chronic malnutrition among children under five years in the Legal Amazon is a positive indication that nutrition policies and food security programs may be having a positive impact. Programs, such as Bolsa Família, and micronutrient supplementation initiatives, such as the distributions of vitamin A and iron, are examples of interventions that may have contributed to this improvement [28,29,30,31,32]. Additionally, the increased coverage of the Unified Health System (SUS) and the strengthening of primary healthcare in the region [33,34,35,36] may have played a crucial role in reducing undernutrition.

The variation in overweight trends among children in the Legal Amazon, with periods of increase and decrease over the last decade, may reflect changes in schools’ feeding policies [37,38,39], economic fluctuations, regional differences in access to ultra-processed foods [40,41,42], and policies restricting the marketing of unhealthy foods to children [43].

The significant increases in overweight among adults and the elderly in the Legal Amazon are worrying trends, as they indicate that the region is undergoing a global nutritional transition characterized by rises in the prevalences of chronic diseases associated with obesity [44,45]. In Brazil, from 1975 to 2019, the prevalences of underweight among adults and the elderly decreased from 9.1% to 2.5% among men and from 12.2% to 3.4% among women. In contrast, during the same period, the prevalences of obesity increased from 3% to 22% among men and from 9% to 30% among women [9]. The elevated prevalence of overweight among the elderly in the Legal Amazon, compared to other regions of Brazil [46], indicates that health policies need to be adapted to better address the needs of this specific population, particularly in rural and less accessible areas. Additionally, there is an increased risk of sarcopenia in older adults with obesity and lower muscle mass, which may raise the occurrences of cardiovascular diseases and associated mortality, as well as metabolic disorders, cognitive impairment, arthritis, functional limitations, and lung diseases [47].

Compared to other tropical regions, the Legal Amazon faces challenges similar to those observed in parts of Asia and Latin America, where rapid urbanization and the expansion of the ultra-processed food market are fueling the rise in obesity [42,45,48,49]. For instance, in India, between 2006 and 2016, the prevalences of underweight among adults decreased significantly in both sexes, from 36% to 23% among women and from 34% to 20% among men. However, during the same period, the prevalences of overweight nearly doubled, rising from 12.5% to 20.5% among women and from 10.1% to 19.1% among men [50].

A recent study has provided updated estimates on the prevalences of undernutrition, obesity, and their interrelationships across different age groups from 1990 to 2022 in countries worldwide. It found that between 1990 and 2022, the prevalences of obesity increased in 81% of the countries for women and 70% for men, while undernutrition decreased in some countries, particularly in South Asia. Among children and adolescents, the combination of underweight and obesity also increased in approximately 70% of the countries. Obesity has surpassed undernutrition in most regions, reflecting a significant transition in dietary patterns and health [51].

On the other hand, although research indicates a reduction in the prevalence of underweight, as measured based on the body mass index (BMI), nutritional deficiencies, characterized by micronutrient deficiencies, persist and are reported in the literature. This is because an inadequate diet or an excess of empty calories can lead to obesity while still causing nutritional deficiencies (hidden hunger), which negatively impact child development, economic stability, and overall public health [52].

In this context, it is important to examine factors that may be associated with the double burden of malnutrition. Changes in Brazilians’ lifestyle habits, associated with urbanization and rising incomes, may have contributed to a reduction in underweight and an increase in overweight over the past decade [8,9,48]. A population study of children and women who participated in national demographic and health surveys in Brazil, Bolivia, Colombia, and Peru revealed prevalences of the double burden of malnutrition of 9.3% in Bolivia, 6.7% in Peru, 3.2% in Colombia, and 2.2% in Brazil [53].

It should be noted that the economic crisis, exacerbated by the COVID-19 pandemic since 2020, has worsened conditions of food and nutritional insecurity for families, especially because of a combination of factors, such as increased unemployment, reduced income, and the rise in prices of essential foods. The COVID-19 pandemic, with its direct impacts on the economy and the labor market, amplified this scenario, leading to greater food vulnerability for many families [54]. This scenario has likely accelerated the consumption of ultra-processed foods, as they are low-cost and easily accessible, contributing to the double burden of diseases associated with overweight and nutritional deficiencies [45].

In addition, environmental changes have impeded the adequate distribution of fresh food. As a result, socioeconomic inequalities, related to difficulties in physical and financial access, have affected a growing number of families, contributing to micronutrient shortages and increasing food insecurity. This situation has led to a rise in undernutrition, while simultaneously contributing to an increase in overweight within the same population, creating a complex scenario of double nutritional burdens [22,55,56].

The findings of this study indicate the urgent need to adapt public health policies to address both undernutrition and the rises in overweight and obesity in the Legal Amazon. It is recommended that existing policies be strengthened, new initiatives be implemented to improve access to nutritious and affordable food (particularly in remote regions), and nutritional education programs aimed at different age groups be expanded [28,30,57]. Furthermore, it is necessary to consider the strengthening of policies that restrict the marketing of ultra-processed foods to children and promote the creation of healthy school environments [58,59,60].

Specific interventions should be developed to address the needs of different age groups [60]. For children, food supplementation programs and nutritional education are essential to continue reducing the prevalence of undernutrition [55]. For adults, it is crucial to promote policies that encourage physical activity and improve diet quality, such as taxation on sugary beverages and the promotion of natural foods—strategies that have been successful in other countries [60,61,62]. For the elderly, strategies should focus on promoting adequate and healthy diets, maintaining an active lifestyle, and ensuring access to appropriate healthcare [63,64].

This study on malnutrition in the Legal Amazon presents several strengths that highlight its relevance. First, it utilizes an extensive database, encompassing data on more than 10 million children, adults, and older adults monitored using SISVAN between 2013 and 2023. This large dataset enables a robust analysis of malnutrition in the region.

Furthermore, this study employs spatial and temporal approaches, extending beyond a mere observation of malnutrition prevalence. It examines trends over time and geographic distribution patterns, which are essential for identifying the most vulnerable areas requiring greater attention. The stratification of the data by age group and sex provides a detailed understanding of the differences in malnutrition prevalence across various population segments, further enriching the analysis.

Another key strength of this research is its proposal of targeted interventions for different age groups, contributing to the development of more effective public policies. Additionally, by considering the cultural diversity and socioeconomic conditions of the Legal Amazon, this study ensures a more accurate interpretation of the data and facilitates the formulation of strategies tailored to the local reality.

These aspects underscore this study’s importance not only in identifying nutritional challenges but also in developing strategies that can positively impact the health and wellbeing of the region’s population.

This study also presents some methodological limitations, particularly concerning the quality and coverage of the SISVAN data. The collection of anthropometric data in remote areas may have been compromised by the limited infrastructure, and variability in data quality across municipalities may have influenced the results. Nonetheless, it is important to note that SISVAN coverage has been progressively expanding in this region [65,66,67]. Moreover, SISVAN has faced several challenges, such as the underutilization of the information generated and the need for the training of professionals to properly collect and analyze data. The limited dissemination of some data presented in this study can also be attributed to the lack of user adherence, which undermines its effectiveness, in addition to the workload overload, particularly among nurses, making it difficult to perform critical analyses and implement effective actions, as shown in other studies that used this system [68,69]. However, despite these challenges, SISVAN is highly regarded by healthcare professionals as essential for primary care, enabling the identification of specific profiles and guiding targeted interventions. The system facilitates the monitoring of nutritional status and integrates the routines of nursing teams, improving the collection of essential data to support the formulation of public policies beyond nutritional health [68]. Therefore, although it faces operational challenges, the benefits of SISVAN in promoting food and nutritional surveillance are indisputable, emphasizing the need for continuous efforts to overcome its specific limitations [69].

A possible selection bias may affect this study because of low user adherence and difficulties in completing the required information, which could lead to underreporting of malnutrition and obesity cases, compromising the assessment of the population’s nutritional status. In this study, interobserver variability among anthropometrists was not assessed; however, the healthcare professionals have experience in anthropometry. Combined with precarious infrastructure, these factors impact data representativeness, resource allocation, and the formulation of adequate public policies, particularly for vulnerable groups, such as indigenous and riverine communities in the Legal Amazon.

It was observed that the national coverage of the program ranges from 9.78% to 14.92%, far below the expected level for a system created to serve the entire country [70]. According to the revised data, the coverage of SISVAN is predominantly concentrated in the maternal–infant group, while the registration of the elderly is infrequently carried out. In studies, coverage among children ranged from 4.3% to 10.7% [71], with averages around 27.7% in some analyses [70]. Although there are no specific data highlighted on the Legal Amazon, the literature suggests that public policies prioritized in this region may have impacted the system’s coverage differently compared to that in other regions [71,72,73,74,75]. Nascimento (2016) indicates that between 2008 and 2013, there were significant variations in coverage, with the north and northeast regions showing increases in their coverage frequencies [70], possibly because of the prioritization of public policies in these regions [76].

The socioeconomic and cultural diversities of the Brazilian population further underscore the need for strategies to ensure the inclusion of these groups in public health statistics, thereby enhancing the accuracy of analyses and the effectiveness of interventions [69].

However, despite these challenges, positive outcomes have been observed in an ongoing study conducted by institutions, such as the Oswaldo Cruz Foundation and various Brazilian universities, aiming to evaluate the quality of anthropometric data from children under five years old monitored using the Food and Nutrition Surveillance System (SISVAN) between 2008 and 2017 [66]. The results indicated a significant improvement in coverage, increasing from 17.7% to 45.4% over the period. The study analyzed multiple indicators, revealing a high level of data completeness and a balanced distribution between the sexes. Nevertheless, concerns were raised regarding the accuracy of height measurements, particularly among vulnerable groups, such as young children and residents of the north and northeast regions, where higher rates of implausible Z-scores were observed [66]. Only 35% of the basic health units in Brazil possess adequate infrastructure, including scales, stadiometers, and anthropometric tapes, essential for collecting reliable anthropometric data for nutritional surveillance. This limitation compromises the accuracy of weight and height measurements, which are fundamental for monitoring nutritional status and evaluating the effectiveness of health interventions [66]. The study concludes that despite progress, continuous efforts must focus on improving data quality and expanding coverage through intersectoral policies and capacity building for healthcare professionals to enhance nutritional monitoring, particularly in the Brazilian pediatric population [66]. Therefore, investing in the improvement of infrastructure and equipment in these units is crucial for ensuring the collection of reliable data. Throughout the study, it became evident that enhancing outcomes requires the development of qualifications and continuous education initiatives for healthcare professionals. This includes comprehensive training to ensure that personnel are adequately prepared to perform precise measurements and accurately record specific information [66].

In Brazil, various manuals and guidelines have been made freely available to train primary care managers and professionals on data collection and tabulation for SISVAN [77,78,79,80,81].

The regional specificities of the Legal Amazon, including its vast territorial extension, environmental diversity, and unequal socioeconomic conditions, must be considered when interpreting the results. The complexity of accessing health services and the variability in social determinants of health across states and municipalities indicate that public policies should be adapted to local realities.

Climate change in the Amazon region has significant impacts on food production by compromising agriculture and livestock because of water scarcity, which can result in yield reductions of up to 60% in certain areas. Changes in the water cycle affect water availability, consequently impacting food security, especially in the face of increased extreme weather events, such as prolonged droughts or heavy rains, which can lead to catastrophic flooding. Furthermore, the degradation of ecosystems undermines essential services for agriculture, such as pollination and soil fertility. Shifts in seasonal patterns and precipitation make agriculture even more unpredictable, primarily affecting smallholder farmers who rely on stable climatic conditions. Together, these factors signal a challenging future for food security in the Amazon region [82].

Future research should focus on further exploring the social and environmental determinants of the double burden of malnutrition in the Legal Amazon. Qualitative studies investigating eating habits, lifestyles, and perceptions of nutrition in the region could offer valuable insights for developing more effective policies. Additionally, it is important to assess the impacts of recent public policies, such as those related to school meals and the regulation of ultra-processed foods, on the prevalences of overweight and obesity.

To improve the understanding of nutritional dynamics in the Legal Amazon, it is recommended to use methodological approaches that integrate both quantitative and qualitative data, as well as longitudinal data, to monitor changes over time. Incorporating social and economic indicators into the analyses can help to identify the underlying determinants of regional inequalities and guide the development of more effective interventions.

## 5. Conclusions

This study observed reductions in the prevalences of chronic malnutrition and overweight in children under five years of age and increases in overweight among adults and the elderly in the Legal Amazon from 2013 to 2023.

Despite the reductions in malnutrition (chronic and acute) prevalences observed in children under five, the prevalences in this region remain predominantly higher than the national averages, and spatial analysis revealed significant regional disparities within the Brazilian Amazon territory. The substantial increases in overweight prevalences among adults and the elderly are also concerning.

In light of these findings, it is concluded that the double burden of malnutrition in the Legal Amazon remains a challenge for this region, necessitating the reinforcement of existing public policies and the development of an intersectoral agenda to address malnutrition in this territory.

## Figures and Tables

**Table 1:** Distributions of prevalence (%) and annual percentage changes (APCs) in malnutrition in children under 5 years of age, stratified by sex, in the Legal Amazon in 2013–2023.

Nutritional Status	2013	2014	2015	2016	2017	2018	2019	2020	2021	2022	2023	APC	95% CI	Period
**Stunting (height-for-age index: Z-score < −2)**														
Total	20.45	20.03	17.82	17.93	18.1	17.99	18.56	17.07	15.79	15.13	15.3	−2.7 *	−3.7; −1.6	2013–2023
Male	21.6	21.74	19.19	19.19	19.4	19.29	20.01	18.41	17.05	16.45	16.6	−2.5 *	−3.6; −1.4	2013–2023
Female	19.51	18.52	16.49	16.62	16.75	16.63	17.04	15.66	14.47	13.74	13.92	−3.0 *	−4.2; −1.8	2013–2023
**Wasting (weight-for-height index: Z-score < −2)**														
Total	6.86	6.35	6.26	6.45	6.45	5.46	5.95	6.32	6.87	6.44	5.84	−0.5	−1.9; 0.9	2013–2023
Male	6.86	6.42	6.36	6.47	6.53	5.6	6.04	6.41	7.03	6.55	5.94	−0.3	−1.8; 1.08	2013–2023
Female	6.89	6.3	6.15	6.42	6.37	5.31	5.96	6.24	6.71	6.33	5.74	−0.60	−1.9; 0.80	2013–2023
**Overweight (weight-for-height index: Z-score > +2)**														
Total	15.23	16.3	13.6	14.89	13.07	12.42	13.23	13.97	14.01	12.81	11.8	−4.7 *	−6.6; −3.7	2013–2018
												4.0 *	1.2; 5.8	2018–2021
												−9.4 *	−13.6; −5.1	2021–2023
Male	16.13	16.96	14.29	15.57	13.77	13.1	13.91	14.75	14.69	13.54	12.48	−4.5 *	−6.4; −3.5	2013–2018
												3.9 *	1.2; 5.7	2018–2021
												−9.1 *	−13.2; −4.8	2021–2023
Female	14.53	15.21	12.91	14.19	12.34	11.72	12.51	13.15	13.29	12.04	11.09	−4.6 *	−6.4; −3.7	2013–2018
												3.9 *	1.2; 5.7	2018–2021
												−9.6 *	−13.5; −5.7	2021–2023
**Obesity (weight-for-height index: Z-score > +3)**														
Total	9.04	9.72	7.15	8.47	6.62	5.77	6.63	6.76	7.2	6.19	5.42	−9.0 *	−11.6; −7.7	2013–2018
												5.5 *	1.2; 8.9	2018–2021
												−13.4 *	−19.2; −7.0	2021–2023
Male	9.65	10.28	7.5	8.9	7.08	6.19	7.14	7.28	7.69	6.7	5.83	−8.7 *	−10.4; −7.7	2013–2018
												5.7 *	1.8; 8.6	2018–2021
												−13.2 *	−17.8; −8.1	2021–2023
Female	8.55	9.23	6.81	8.02	6.14	5.33	6.1	6.21	6.69	5.64	4.99	−9.6 *	−11.5; −8.6	2013–2018
												5.4 *	1.2; 8.5	2018–2021
												−13.6 *	−18.6; −7.9	2021–2023

* Significantly different from zero at the alpha level = 0.05.

**Table 2:** Distributions of prevalence (%) and annual percentage changes (APCs) in malnutrition in adults, stratified by sex, in the Legal Amazon in 2013–2023.

Nutritional Status	2013	2014	2015	2016	2017	2018	2019	2020	2021	2022	2023	APC	95% CI	Period
**Underweight (BMI < 18.5 kg/m^2^)**														
Total	3.87	3.66	2.86	2.99	2.64	2.48	2.63	2.23	2.17	2.27	2.26	−13.5 *	−15.8; −9.4	2013–2015
												−5.2 *	−6.7; −3.9	2015–2021
												2.2	−2.9; 5.2	2021–2023
Male	3.77	3.65	2.09	2.05	1.85	1.77	1.76	1.74	1.76	1.96	2.01	−22.8 *	−29.8; −19.1	2013–2016
												−2.4	−9.7; 2.0	2016–2020
												5.9 *	1.5; 13.4	2020–2023
Female	3.87	3.66	2.92	3.09	2.71	2.55	2.74	2.36	2.29	2.38	2.35	−12.4 *	−14.7; −8.7	2013–2015
												−4.6 *	−5.9; −3.5	2015–2021
												1.4	−2.9; 4.3	2021–2023
**Overweight (BMI ≥ 25.0 kg/m^2^)**														
Total	48.85	51.1	55.04	55,00	56.33	58.69	59.75	62.88	63.87	63.56	64.64	5.1 *	3.2; 7.1	2013–2015
												2.8 *	2.1; 3.5	2015–2021
												0.4	−1.5; 2.3	2021–2023
Male	49.1	50.1	57.51	57.71	58.16	58.85	58.33	60.62	61.53	60.42	60.68	9.5 *	5.8; 12.1	2013–2015
												1.0 *	0.4; 1.3	2015–2023
Female	48.84	51.11	54.87	54.7	56.17	58.68	59.93	63.49	64.56	64.68	66.17	3.1 *	2.7; 3.3	2013–2023
**Obesity (BMI ≥ 30.0 kg/m^2^)**														
Total	17.1	18.4	20.67	20.88	21.61	23.06	24.05	26.27	27.32	27.34	28.49	9.2 *	7.0; 11.9	2013–2015
												3.9 *	2.7; 5.2	2015–2018
												6.5 *	5.2; 7.7	2018–2021
												1.1	−1.3; 3.1	2021–2023
Male	14.47	14.64	19.66	19.73	20,00	20.29	19.9	21.78	23.08	22.41	23.02	18.3 *	8.3; 28.1	2013–2015
												2.6 *	0.5; 3.6	2015–2023
Female	17.13	18.44	20.74	21	21.76	23.33	24.58	27.48	28.57	29.09	30.61	5.9 *	5.3; 6.6	2013–2023

* Significantly different from zero at the alpha level = 0.05.

**Table 3:** Distributions of prevalence (%) and annual percentage changes (APCs) in malnutrition in elderly, stratified by sex, in the Legal Amazon in 2013–2023.

Nutritional Status	2013	2014	2015	2016	2017	2018	2019	2020	2021	2022	2023	APC	95% CI	Period
**Underweight (BMI < 22.0 kg/m^2^)**														
Total	17.47	17.93	14.68	14.63	13.93	13.05	12.95	12.95	12.69	12.9	13.04	−6.3 *	−8.3; −5.2	2013–2018
												−0.0	−1.5; 2.3	2018–2023
Male	19.3	19.32	14.98	15.12	14.53	13.73	13.63	13.45	13.15	13.45	13.76	−10.8 *	−16.8; −6.8	2013–2016
												−1.3	−2.5; 0.8	2016–2023
Female	16.26	17.72	14.52	14.31	13.55	12.62	12.52	12.55	12.34	12.45	12.47	−6.3 *	−7.7; −5.3	2013–2018
												−0.3	−1.5; 1.5	2018–2023
**Overweight (BMI ≥ 27.0 kg/m^2^)**														
Total	42.35	41.96	45.78	45.84	46.92	48.31	48.23	47.48	47.75	47.97	48.22	3.4 *	2.5; 5.1	2013–2017
												0.1	−0.7; 0.6	2017–2023
Male	36.03	35.74	41.23	40.9	41.79	42.7	42.6	42.65	42.79	43.03	43.13	6.1 *	4.7; 8.2	2013–2016
												0.5 *	0.1; 0.8	2016–2023
Female	46.51	42.88	48.23	48.99	50.15	51.83	51.82	51.26	51.61	51.89	52.31	3.2 *	2.3; 5.1	2013–2018
												0, 0	−1.8; 0.9	2018–2023

* Significantly different from zero at the alpha level = 0.05.

## Data Availability

The data presented in this study are publicly and unrestrictedly available from the Brazilian Ministry of Health on the website of the SISVAN, https://sisaps.saude.gov.br/sisvan/ (accessed on 5 July 2024).

## Supplementary Materials

The following supporting information can be downloaded at: https://www.mdpi.com/article/10.3390/nu17061054/s1, Table S1: Distributions of prevalence (%) and annual percentage changes (APCs) in stunting based on the height-for-age index (Z-score < −2) in children under 5 years old, stratified by state and sex, in the Legal Amazon in 2013–2023; Table S2: Distributions of prevalence (%) and annual percentage changes (APCs) in wasting based on the weight-for-height index (Z-score < −2) in children under 5 years old, stratified by state and sex, in the Legal Amazon in 2013–2023; Table S3: Distributions of prevalence (%) and annual percentage changes (APCs) in overweight based on the weight-for-height index (Z-score > +2) in children under 5 years old, stratified by state and sex, in the Legal Amazon in 2013–2023; Table S4: Distributions of prevalence (%) and annual percentage changes (APCs) in obesity based on the weight-for-height index (Z-score > +3) in children under 5 years old, stratified by state and sex, in the Legal Amazon in 2013–2023; Table S5: Distributions of prevalence (%) and annual percentage changes (APCs) in underweight (BMI < 18.5 kg/m^2^) in adults, stratified by state and sex, in the Legal Amazon in 2013–2023; Table S6: Distributions of prevalence (%) and annual percentage changes (APCs) in overweight (BMI ≥ 25.0 kg/m^2^) in adults, stratified by state and sex, in the Legal Amazon in 2013–2023; Table S7: Distributions of prevalence (%) and annual percentage changes (APCs) in obesity (BMI ≥ 30.0 kg/m^2^) in adults, stratified by state and sex, in the Legal Amazon in 2013–2023; Table S8: Distributions of prevalence (%) and annual percentage changes (APCs) in underweight (BMI < 22.0 kg/m^2^) in older adults, stratified by state and sex, in the Legal Amazon in 2013–2023; Table S9: Distributions of prevalence (%) and annual percentage changes (APCs) in overweight (BMI ≥ 27.0 kg/m^2^) in older adults, stratified by state and sex, in the Legal Amazon in 2013–2023.

## References

[1] World Health Organization (2021). Fact Sheets: Malnutrition. https://www.who.int/news-room/fact-sheets/detail/malnutrition.

[2] Popkin B.M., Reardon T. (2018). Obesity and the food system transformation in Latin America. Obes. Rev..

[3] World Health Organization (2024). Malnutrition. https://www.who.int/news-room/questions-and-answers/item/malnutrition.

[4] Agência Brasil Desnutrição no Brasil é Maior Entre Meninos Negros, Aponta Pesquisa. https://agenciabrasil.ebc.com.br/saude/noticia/2022-07/desnutricao-no-brasil-e-maior-entre-meninos-negros-aponta-pesquisa.

[5] Ministério da Saúde Pesquisa do IBGE Mostra Aumento da Obesidade Entre Adultos. https://www.gov.br/pt-br/noticias/saude-e-vigilancia-sanitaria/2020/10/pesquisa-do-ibge-mostra-aumento-da-obesidade-entre-adultos.

[6] Malta D.C., Gomes C.S., Veloso G.A., Souza J.B.d., Oliveira P.P.V.d., Ferreira A.V.L., Nagavi M., Ferrinho P., Freitas P.C.d., Ribeiro A.L.P. (2023). Carga Das Doenças Crônicas Não Transmissíveis Nos Países De Língua Portuguesa. Ciência Saúde Coletiva.

[7] Rivera J.A., Pedraza L.S., Martorell R., Gil A. (2014). Introduction to the double burden of undernutrition and excess weight in Latin America. Am. J. Clin. Nutr..

[8] Meller F.O., Schäfer A.A., Santos L.P., Quadra M.R., Miranda V.I.A. (2021). Double Burden of Malnutrition and Inequalities in the Nutritional Status of Adults: A Population-Based Study in Brazil, 2019. Int. J. Public Health.

[9] Conde W.L., Silva I.V.d., Ferraz F.R. (2022). Tendências de desnutrição e obesidade em adultos brasileiros de 1975 a 2019 e seus fatores associados. Cad. Saude Publica.

[10] Guerra L.D.d.S., Vieira Passos E. (2023). A fome como arma de extermínio: O genocídio alimentar e nutricional dos povos indígenas Xavante na Amazônia Legal Brasileira. J. Manag. Prim. Health Care.

[11] Malta D.C., Gomes C.S., Felisbino-Mendes M.S., Veloso G.A., Machado I.E., Cardoso L.d.O., Azeredo R.T., Jaime P.C., de Vasconcelos L.L.C., Naghavi M. (2024). Undernutrition, and overweight and obesity: The two faces of malnutrition in Brazil, analysis of the Global Burden of Disease, 1990 to 2019. Public Health.

[12] Popkin B.M., Corvalan C., Grummer-Strawn L.M. (2020). Dynamics of the double burden of malnutrition and the changing nutrition reality. Lancet.

[13] Nugent R., Levin C., Hale J., Hutchinson B. (2020). Economic effects of the double burden of malnutrition. Lancet.

[14] Dias F.S.B., Silva T.F.D., Lima Y.d.M.M., Farias L.S.D., Gadelha J.G., Ramalho A.A. (2023). Temporal Trend of Severe Obesity in Brazilian State Capitals (2006–2021). Obesities.

[15] Instituto Brasileiro de Geografia e Estatística (2022). Cadastro de Municípios Localizados na Amazônia Legal. https://www.ibge.gov.br/geociencias/informacoes-ambientais/vegetacao/15819-amazonia-legal.html?t=o-que-e.

[16] World Health Organization (2006). Who Child Growth Standards: Length/Height-for-Age, Weight-for-Age, Weightfor-Length, Weight-for-Height and Body Massindex-for-Age: Methods and Development.

[17] World Health Organization (2000). Obesity: Preventing and Managing the Global Epidemic: Report of a WHO Consultation.

[18] Lipschitz D.A. (1994). Screening for nutritional status in the elderly. Prim. Care.

[19] World Health Organization (1995). Physical Status: The Use and Interpretation of Anthropometry.

[20] Kim H.J., Fay M.P., Feuer E.J., Midthune D.N. (2000). Permutation Tests for Joinpoint Regression with Applications to Cancer Rates. Stat. Med..

[21] World Health Organization (2016). The Double Burden of Malnutrition: Policy Brief.

[22] Swinburn B.A., Kraak V.I., Allender S., Atkins V.J., Baker P.I., Bogard J.R., Dietz W.H. (2019). The global syndemic of obesity, undernutrition, and climate change: The Lancet Commission report. Lancet.

[23] Dukhi N. (2020). Global prevalence of malnutrition: Evidence from literature. Malnutrition.

[24] World Health Organization (2020). Obesity and Overweight. https://www.who.int/news-room/fact-sheets/detail/obesity-and-overweight.

[25] Ribeiro-Silva R.d.C., Silva N.d.J., Felisbino-Mendes M.S., Falcão I.R., de Andrade R.d.C.S., Silva S.A. (2022). Time trends and social inequalities in child malnutrition: Nationwide estimates from Brazil’s food and nutrition surveillance system, 2009–2017. Public Health Nutr..

[26] Stopa S.R., Malta D.C., Monteiro C.N., Szwarcwald C.L., Goldbaum M., Cesar C.L.G. (2017). Acesso e uso de serviços de saúde pela população brasileira, Pesquisa Nacional de Saúde 2013. Rev. Saude Publica.

[27] Palmeira N.C., Moro J.P., Getulino F.A., Vieira Y.P., Junior A.O.S., Saes M.O. (2022). Análise do acesso a serviços de saúde no Brasil segundo perfil sociodemográfico: Pesquisa Nacional de Saúde, 2019. Epidemiol. Serv. Saúde.

[28] Bortolini G.A., Pereira T.N., Nilson E.A.F., Pires A.C.L., Moratori M.F., Ramos M.K.P. (2021). Evolution of nutrition actions in primary health care along the 20-year history of the Brazilian National Food and Nutrition Policy. Cad. Saúde Pública.

[29] Alves K.P.S. (2021). Enfrentamento das carências de micronutrientes no Brasil: Reflexões sobre as estratégiaS no âmbito da Atenção Básica. Alimentação e Nutrição na Atenção Básica: Reflexões Cotidianas e Contribuições para Prática do Cuidado.

[30] Bortolini G.A., Oliveira T.F.V., Silva S.A., Santin R.C., Medeiros O.L., Spaniol A.M. (2020). Ações de alimentação e nutrição na atenção primária à saúde no Brasil. Rev. Panam. Salud Publica.

[31] Souza A.A.d., Heller L. (2021). Bolsa Família Program and environmental health: A systematic review of the effects on diarrhea and malnutrition. Ciência Saúde Coletiva.

[32] Moriguchi W.L., Bernardes P.D.H., Pinhel A.S.M., Noronha N.Y., Diani M.L., Cintra P.A.L., Ferreira N.C., Barbosa N.C. (2022). Políticas Públicas de Alimentação e Nutrição no Brasil: Da Desnutrição à Obesidade. Nutrientes.

[33] Castro M.C., Massuda A., Almeida G., Menezes-Filho N.A., Andrade M.V., Souza Noronha K.V.M., Rocha R., Macinko J., Hone T., Tasca R. (2019). Brazil’s unified health system: The first 30 years and prospects for the future. Lancet.

[34] Carneiro V.C.C.B., Ribeiro d.O.P.T., Rassy C.S., Cardoso M.M., Pedroso J.D.S. (2022). Impact of expansion of primary care in child health: A population-based panel study in municipalities in the Brazilian Amazon. BMJ Open.

[35] Coube M., Nikoloski Z., Mrejen M., Mossialos E. (2023). Persistent inequalities in health care services utilisation in Brazil (1998–2019). Int. J. Equity Health.

[36] Viacava F., Oliveira R.A.D.D., Carvalho C.D.C., Laguardia J., Bellido J.G. (2018). SUS: Supply, access to and use of health services over the last 30 years. Ciência Saúde Coletiva.

[37] Canella D.S., Bandeira L., Oliveira M.L., Castro S., Pereira A.D.S., Bandoni D.H., Castro I.R.R. (2022). Update of the acquisition parameters of the Brazilian National School Feeding Program based on the Dietary Guidelines for the Brazilian Population. Cad. Saude Publica.

[38] Tuliende M.I.E.D., Martinelli S.S., Soares P., Fabri R.K., Bianchini V.U., Cavalli S.B. (2024). Benefits and Difficulties of Implementing Family-Farming Food Purchases in the Brazilian National School Feeding Program. Int. J. Public Health.

[39] Nero D.d.S.M., Garcia R.P.M., Almassy Junior A.A. (2023). O Programa Nacional de Alimentação Escolar (Pnae) a partir da sua gestão de descentralização. Ens. Avaliação Políticas Públicas Educ..

[40] Claro R.M., Maia E.G., Costa B.V., Diniz D.P. (2016). Preço dos alimentos no Brasil: Prefira preparações culinárias a alimentos ultraprocessados. Cad. Saude Publica.

[41] Da Silva Oliveira E.K., Vieira T.D.S., de Souza O.F., Bezerra I.M.P., Cavalcanti M.P.E., de Abreu L.C., Riera A.R.P. (2024). Consumption of Ultra-Processed Foods in the Brazilian Amazon during COVID-19. Nutrients.

[42] Passos C.M.D., Maia E.G., Levy R.B., Martins A.P.B., Claro R.M. (2020). Association between the price of ultra-processed foods and obesity in Brazil. Nutr. Metab. Cardiovasc. Dis..

[43] Carvalho C.M.P., Johns P., Albiero M., Martins A.P.B., Mais L.A., Ralston R., Collin J. (2022). “Private and personal”: Corporate political activity, informal governance, and the undermining of marketing regulation in Brazil. Glob. Public Health.

[44] Popkin B.M., Shu W. (2022). The nutrition transition to a stage of high obesity and noncommunicable disease prevalence dominated by ultra-processed foods is not inevitable. Obes. Rev..

[45] Food and Agriculture Organization of the United Nations (2024). The State of Food Security and Nutrition in the World 2024—Financing to End Hunger, Food Insecurity and Malnutrition in All Its Forms.

[46] Rodrigues L.C., Canella D.S., Claro R.M. (2021). Time trend of overweight and obesity prevalence among older people in Brazilian State Capitals and the Federal District from 2006 to 2019. Eur. J. Ageing.

[47] Liu C., Wong P.Y., Chung Y.L., Chow S.K.-H., Cheung W.H., Law S.W., Chan J.C.N., Wong R.M.Y. (2023). Deciphering the “obesity paradox” in the elderly: A systematic review and meta-analysis of sarcopenic obesity. Obes. Rev..

[48] Baker P., Machado P., Santos T., Sievert K., Backholer K., Hadjikakou M., Russell C., Huse O., Bell C., Scrinis G. (2020). Ultra-processed foods and the nutrition transition: Global, regional and national trends, food systems transformations and political economy drivers. Obes. Rev..

[49] Levy R.B., Barata M.F., Leite M.A., Andrade G.C. (2024). How and why ultra-processed foods harm human health. Proc. Nutr. Soc..

[50] Nguyen P.H., Scott S., Headey D., Singh N., Tran L.M. (2021). The double burden of malnutrition in India: Trends and inequalities (2006–2016). PLoS ONE.

[51] NCD Risk Factor Collaboration (NCD-RisC) (2024). Worldwide trends in underweight and obesity from 1990 to 2022: A pooled analysis of 3663 population-representative studies with 222 million children, adolescents, and adults. Lancet.

[52] Lopes S.O., Abrantes L.C.S., Azevedo F.M., Morais N.d.S., Morais D.d.C., Gonçalves V.S.S., Fontes E.A.F., Franceschini S.d.C.C., Priore S.E. (2023). Insegurança alimentar e deficiência de micronutrientes em adultos: Uma revisão sistemática e meta-análise. Nutrients.

[53] Temponi H.R., Velasquez-Melendez G. (2020). Prevalence of double burden on malnutrition at household level in four Latin America countries. Rev. Bras. Saúde Matern. Infant..

[54] Salles-Costa R., Segall-Corrêa A.M., Alexandre-Weiss V.P., Pasquim E.M., Paula N.M.d., Lignani J.d.B., Grossi M.E.D., Zimmermann S.A., Medeiros M.A.T.d., Santos S.M.C.d. (2023). Rise and fall of household food security in Brazil, 2004 to 2022. Cad. Saúde Pública.

[55] Kasperson J.X., Kasperson R.E., Turner B.L., Hsieh W., Schiller A. (2022). Vulnerability to global environmental change. The Social Contours of Risk.

[56] Brandão A.L., Casemiro J.P., Peres F. (2024). Insegurança Alimentar e Emergência Climática: Sindemia Global e um Desafio de Saúde Pública na América Latina.

[57] Brandão A.L., Casemiro J.P., dos Reis E.C., Vitorino S.A.S., de Oliveira A.D.S.B., Bortolini G.A. (2022). Recomendações para o fortalecimento da atenção nutricional na atenção primária à saúde brasileira. Rev. Panam. Salud Publica.

[58] Taillie L.S., Busey E., Stoltze F.M., Dillman Carpentier F.R. (2019). Governmental policies to reduce unhealthy food marketing to children. Nutr. Rev..

[59] World Health Organization (2023). Policies to Protect Children from the Harmful Impact of Food Marketing: WHO Guideline.

[60] Pereira T.N., Bortolini G.A., Campos R.d.F. (2023). Barriers and Facilitators Related to the Adoption of Policies to Reduce Ultra-Processed Foods Consumption: A Scoping Review. Int. J. Environ. Res. Public Health.

[61] Smith E., Scarborough P., Rayner M., Briggs A.D.M. (2018). Should we tax unhealthy food and drink?. Proc. Nutr. Soc..

[62] Popkin B.M., Barquera S., Corvalan C., Hofman K.J., Monteiro C., Ng S.W., Swart E.C., Taillie L.S. (2021). Towards unified and impactful policies to reduce ultra-processed food consumption and promote healthier eating. Lancet Diabetes Endocrinol..

[63] World Health Organization (2021). Decade of Healthy Ageing: Baseline Report. https://www.who.int/publications/i/item/9789240017900.

[64] Keating N. (2022). A research framework for the United Nations Decade of Healthy Ageing (2021–2030). Eur. J. Ageing.

[65] Silva R.P.C., Vergara C.M.A.C., Sampaio H.A.C., Vasconcelos Filho J.E., Strozberg F., Ferreira Neto J.F.R., Mafra M.L.P., Garcia Filho C., Carioca Filho A.A.F. (2022). Food and Nutrition Surveillance System: Temporal trend of coverage and nutritional status of adults registered on the system, Brazil, 2008-2019. Epidemiol. Serv. Saude.

[66] Silva N.d.J., Mello e Silva J.F.d., Carrilho T.R.B., Pinto E.d.J., Andrade R.d.C.S.d., Silva S.A., Pedroso J., Spaniol A.M., Bortolini G.A., Fagundes A. (2023). Quality of child anthropometric data from SISVAN, Brazil, 2008–2017. Rev. Saúde Pública.

[67] Barbosa B.B., Baltar V.T., Horta R.L., Lobato J.C.P., Vieira L.J.E.S., Gallo C.O., Carioca A.A.F. (2023). Food and Nutrition Surveillance System (SISVAN) coverage, nutritional status of older adults and its relationship with social inequalities in Brazil, 2008-2019: An ecological time-series study. Epidemiol. Serv. Saude.

[68] Alves I.C.R., Souza T.F.d., Leite M.T.S., Pinho L.d. (2018). Limites e possibilidades do Sistema de Vigilância Alimentar e Nutricional na Atenção Primária à Saúde: Relatos de profissionais de enfermagem. DEMETRA Aliment. Nutr. Saúde.

[69] Rolim M.D., Lima S.M.L., Barros D.C.d., Andrade C.L.T.d. (2015). Avaliação Do SISVAN Na Gestão De Ações De Alimentação E Nutrição Em Minas Gerais, Brasil. Ciência Saúde Coletiva.

[70] Nascimento F.A. (2016). A vigilância alimentar e nutricional brasileira na produção científica e nos serviços de saúde. Master’s Thesis.

[71] Ferreira C.S., Souza A.M., Nascimento F.A., Silva R.F. (2013). O sistema de vigilância alimentar e nutricional como instrumento de monitoramento da Estratégia Nacional para Alimentação Complementar Saudável. Rev. Bras. Saúde Mater. Infant..

[72] Pantoja L.N., Narazé L.A., Santos D.F., Costa M.M. (2014). Cobertura do Sistema de Vigilância Alimentar e Nutricional Indígena (SISVAN-I) e prevalência de desvios nutricionais em crianças Yanomami menores de 60 meses, Amazônia, Brasil. Rev. Bras. Saúde Mater. Infant..

[73] Enes C.C., Ferreira A.M., Lima J.S., Pires M.C., Santos F., Morais A., Freitas A.M. (2014). Cobertura populacional do Sistema de Vigilância Alimentar e Nutricional no Estado de São Paulo, Brasil. Ciência Saúde Coletiva.

[74] Jung N.M., Oliveira M.R., Pinto R.M., Silva D.P., Lima C.S. (2014). Utilização e cobertura do Sistema de Vigilância Alimentar e Nutricional no Estado do Rio Grande do Sul, Brasil. Ciência Saúde Coletiva.

[75] Perez A.I., Cruz A.I., Costa N.M., Silva J.B., Barbosa F.M. (2013). Monitoramento do estado nutricional de usuários de Unidades Básicas de Saúde no Estado de São Paulo por meio do Sistema de Vigilância Alimentar e Nutricional (SISVAN). BEPA Bol. Epidemiológico Paul..

[76] Lima J.F., Schmidt D.B., Silva P.F. (2022). Sistema de Vigilância Alimentar e Nutricional: Utilização e Cobertura na Atenção Primária. Atenção Primária à Saúde no Brasil: Avanços, Retrocessos e Práticas em Pesquisa.

[77] Ministério da Saúde (2015). Secretaria de Atenção à Saúde. Departamento de Atenção Básica. Marco de referência da vigilância alimentar e nutricional na atenção básica.

[78] Barros D.C., Silva D.O., Gugelmin S.Â. (2007). Vigilância Alimentar e Nutricional para a Saúde Indígena.

[79] Ministério da Saúde (2017). Manual Operacional para Uso do Sistema de Vigilância Alimentar e Nutricional SISVAN—Versão 3.0.

[80] Ministério da Saúde (2012). Manual Orientador para Aquisição de Equipamentos Antropométricos.

[81] Ministério da Saúde (2022). Guia para a Organização da Vigilância Alimentar e Nutricional na Atenção Primária à Saúde.

[82] Marengo J.A., Souza C. (2018). *Mudanças Climáticas: Impactos e Cenários para a Amazônia*; São Paulo, Brasil. https://prioridadeabsoluta.org.br/wp-content/uploads/2019/05/relatorio_mudancas_climaticas-amazonia.pdf.

