# Supplementary material for: The Double Burden of Malnutrition in the Brazilian Legal Amazon: Spatial Distributions and Temporal Trends (2013–2023)
Source: Nutrients. 2025 Mar 17;17(6):1054. doi: 10.3390/nu17061054 (PMC11945400; doi:10.3390/nu17061054)
Supplement: Supplementary file 1 [file nutrients-17-01054-s001.zip › nutrients-3511476-supplementary.pdf]

Table S1. Distribution of prevalence (%) and annual percentage change (APC) of stunting based on height-for-age index (Z-score < -2) in children under 5 years old by state and sex. Legal Amazon, 2013-2023.

**Variables**	**2013**	**2014**	**2015**	**2016**	**2017**	**2018**	**2019**	**2020**	**2021**	**2022**	**2023**	**APC**	**95% CI**
Legal Amazon	20.45	20.03	17.82	17.93	18.1	17.99	18.56	17.07	15.79	15.13	15.3	-2.7*	-3.7;-1.6
Acre	21.29	22.92	18.95	19.74	17.86	18.48	19.63	18.27	18.02	17.27	17.57	-2.1*	-3.5;-0.7
Amazonas	23.59	22.69	19.79	20.05	21.04	20.13	21.78	19.56	17.35	16.77	17.49	-2.8*	-3.9;-1.6
Amapá	22.24	25.17	21.29	22.34	22.68	22.97	22.21	19.44	18.38	17.5	17.48	-0.5	-2.3;4.4
												-5.9*	-11.5;-4.1
Pará	23.34	20.92	18.31	18.71	19.37	19.22	19.33	17.15	15.68	15.69	16.01	-11.8*	-14.1;-9.5
												2.9*	1.4;4.4
												-7.3*	-8.6;-5.8
												-0.1	-2.9;2.5
Rondônia	11.3	11.18	12.11	9.48	10.48	11.39	12.31	9.39	9.26	9.4	9.42	-2.01*	-3.9;-0.2
Roraima	27.53	26.98	16.88	16.53	17.75	17.83	16.09	13.25	12.65	12.51	14.18	-21.8*	-32.1;-2.9
												-4.9	-20.4;10.2
Tocantins	14.64	14.97	14.03	14.1	13.25	14.36	13.8	13.72	12.89	12.88	13.03	-1.3*	-1.9;-0.7
Maranhão**	19.56	20.23	19.12	19.5	18.62	18.35	18.75	18.43	17.14	17.04	15.98	-1.3*	-1.7;-0.1
												-4.0*	-7.3;-2.5
Mato Grosso	11.67	12.83	11.67	11.86	11.79	11.72	12.85	11.38	12.24	10.01	11.17	-1.1*	-2.2;-0.1
	20.45	20.03	17.82	17.93	18.1	17.99	18.56	17.07	15.79	15.13	15.3	-2.7*	-3.7;-1.6
Male	2013	2014	2015	2016	2017	2018	2019	2020	2021	2022	2023	APC	95% CI
Legal Amazon	21.6	21.74	19.19	19.19	19.4	19.29	20.01	18.41	17.05	16.45	16.6	-2.5*	-3.6;-1.4
Acre	22.5	24.7	20.1	21.09	19.3	20.05	21.03	19.81	19.37	18.69	18.84	-2.0*	-3.3;-0.7
Amazonas	24.32	23.9	20.93	21.41	22.36	21.44	23.3	21.06	18.73	18.26	18.93	-2.4*	-3.6;-1.1
Amapá	23.38	26.06	22.48	23.06	23.54	23.94	23.79	20.99	19.65	18.72	18.62	-2.8*	-4.6;-1.1
Pará	24.51	22.77	19.63	20.05	20.7	20.55	20.78	18.38	16.87	17,00	17.38	-11.2*	-13.6;-8.9
												2.6*	1.0;4.1
												-7.0*	-8.4;-5.5
												0.4	-2.5;3.1
Rondônia	12.15	12.29	13.62	10.18	11.02	12.25	13.17	10.17	10.08	10.26	10.35	-2.1*	-3.9;-0.2
Roraima	29.09	29.14	18.3	17.59	19.08	19.11	17.05	14.83	13.43	13.88	15.39	-21.6*	-32.4;-1.4
												-4.6	-20.7;10.9
Tocantins	15.75	16.54	15.52	15.46	14.62	15.96	15.2	14.86	14.32	14.3	14.32	-1.3*	-1.8;-0.7
Maranhão**	20.95	22.25	20.71	20.83	20.1	19.74	20.4	19.9	18.58	18.52	17.4	-1.9*	-2.6;-1.2
Mato Grosso	12.75	14.1	12.93	12.76	12.75	12.59	13.92	12.42	13.08	10.83	12.15	-1.3*	-2.4;-0.2
Female	2013	2014	2015	2016	2017	2018	2019	2020	2021	2022	2023	APC	95% CI
Legal Amazon	19.51	18.52	16.49	16.62	16.75	16.63	17.04	15.66	14.47	13.74	13.92	-3.0*	-4.2;-1.8
Acre	20.22	21.21	17.79	18.34	16.35	16.83	18.15	16.61	16.57	15.78	16.23	-2.4*	-3.7;-1.0
Amazonas	23.01	21.61	18.69	18.64	19.69	18.77	20.18	17.99	15.9	15.19	15.98	-3.3*	-4.4;-2.2
Amapá	21.26	24.34	20.13	21.61	21.8	21.98	20.56	17.78	17.04	16.2	16.27	-0.9	-2.9;-5.0
												-6.5*	-13.6;-4.5
Pará	22.38	19.26	17.01	17.32	18,00	17.82	17.81	15.82	14.41	14.28	14.56	-13.0*	-15.1;-10.7
												3.1*	1.6;4.5
												-7.6*	-8.9;-6.2
												-0.7	-3.4;1.8
Rondônia	10.64	10.24	10.68	8.74	9.9	10.5	11.41	8.57	8.37	8.48	8.42	-2.3*	-4.2;-0.4
Roraima	26.32	25.22	15.55	15.42	16.33	16.47	15.05	11.58	11.81	11.06	12.95	-26.9*	-32.2;-20.2
												0.7	-3.7;5.4
												-12.5*	-16.4;-8.4
												6.6	-3.1;15.6
Tocantins	13.71	13.57	12.59	12.66	11.83	12.68	12.33	11.59	11.39	11.35	11.65	-1.8*	-2.7;-0.9
Maranhão**	18.46	18.49	17.59	18.1	17.06	16.89	17.02	16.89	15.62	15.49	14.51	-1.5*	-1.9;-0.5
												-4.1*	-7.3;-2.7
Mato Grosso	10.79	11.75	10.45	10.94	10.77	10.82	11.72	10.71	11.34	9.14	10.12	0.2	-0.6;2.3
												-5.4*	-12.1;-2.1

* Significantly different from zero at the alpha level = 0.05.

** Only municipalities in the Legal Amazon.

Table S2. Distribution of prevalence (%) and annual percentage change (APC) of wasting based on weight-for-height index (Z-score < -2) in children under 5 years old by state and sex. Legal Amazon, 2013-2023.

**Variables**	**2013**	**2014**	**2015**	**2016**	**2017**	**2018**	**2019**	**2020**	**2021**	**2022**	**2023**	**APC**	**95% CI**	**Period**
Legal Amazon	6.86	6.35	6.26	6.45	6.45	5.46	5.95	6.32	6.87	6.44	5.84	-0.5	-1.9;0.9	2013-2023
Acre	5.72	5.95	5.74	5.40	5.09	4.57	5.37	4.86	5.76	5.57	4.96	-4.1*	-11.1;-1.6	2013-2018
												2.4	-0.5;-9.6	2018-2023
Amazonas	5.43	5.56	5.11	5.7	7.21	5.15	5.17	5.39	5.82	5.09	4.63	-1.0	-3.5;1.4	2013-2023
Amapá	5.09	4.68	4.64	4.73	4.74	4.41	4.12	4.19	5.09	4.92	4.13	-0.9	-3.2;1.3	2013-2023
Pará	6.68	5.83	6.21	6.11	5.79	5.15	5.8	6.2	6.37	5.95	5.41	-0.5	-2.5;1.4	2013-2023
Rondônia	5.51	5.2	5.38	5.57	5.75	5.32	5.61	6.95	6.1	5.67	5.34	1.1	-0.6;2.7	2013-2023
Roraima	7.48	5.2	4.5	4.3	3.15	3.4	3.5	3.36	3.52	3.37	3.37	-15.9*	-20.5;-12.9	2013-2023
Tocantins	7.13	6.66	6.38	6.19	6.49	4.88	5.36	5.99	6.81	5.4	5.46	-1.9	-3.8;0.01	2013-2023
Maranhão **	8.62	8.03	7.89	8.61	8.05	7.07	7.71	8.17	9.54	10.12	8.96	1.4	-1.0;3.8	2013-2023
Mato Grosso	5.59	5.8	5.04	5.03	4.74	4.28	5.05	5.98	6.03	5.05	5.15	-5.0*	-15.7;-1.4	2013-2018
												11.2*	3.7;16.5	2018-2021
												-11.4*	-21.7;-1.0	2021-2023
Male	2013	2014	2015	2016	2017	2018	2019	2020	2021	2022	2023	APC	95% CI	Period
Legal Amazon	6.86	6.42	6.36	6.47	6.53	5.6	6.04	6.41	7.03	6.55	5.94	-0.3	-1.8;1.08	2013-2023
Acre	5.53	5.83	5.96	5.24	5.05	4.55	5.17	4.99	6.15	5.54	4.81	2.3	-2.04;7.5	2013-2015
												-8.1*	-10.3;-5.7	2015-2018
												8.9*	6.2;11.7	2018-2021
												-8.3*	-12.9;-4.3	2021-2023
Amazonas	5.53	5.72	5.25	5.84	7.34	5.34	5.26	5.48	6,00	5.23	4.72	-1.1	-3.4;1.4	2013-2023
Amapá	4.78	4.95	4.60	4.51	5.13	4.63	4.30	4.09	5.05	4.95	4.23	-0.6	-2.9;1.7	2013-2023
Pará	6.71	5.86	6.38	6.14	5.83	5.31	5.88	6.19	6.44	6.06	5.54	-3.1*	-5.2;-2.1	2013-2018
												5.2*	2.3;7.3	2018-2021
												-7.2	-10.8;-3.1	2021-2023
Rondônia	5.37	4.91	5.42	5.34	5.74	5.30	5.60	7.09	6.45	5.72	5.53	1.9*	0.17;3.6	2013-2023
Roraima	7.79	5.51	4.77	4.32	3.13	3.43	5.73	3.22	3.46	3.43	3.50	-5.8*	-9.6;-1.8	2013-2023
Tocantins	7.09	6.65	6.44	6.2	6.69	4.91	5.26	6,00	6.83	5.50	5.62	-1.7	-3.7;0.1	2013-2023
Maranhão **	8.56	8.15	7.82	8.57	8.14	7.20	7.86	8.36	9.81	10.23	9.02	1.6	-0.9;4.3	2013-2023
Mato Grosso	5.74	5.87	5.1	5.17	4.79	4.43	5.18	6.18	6.19	5.2	5.27	-4.8*	-16.3;-1.0	2013-2018
												11.4*	3.7;17.04	2018-2021
												-11.7*	-22.4;-0.9	2021-2023
Female	2013	2014	2015	2016	2017	2018	2019	2020	2021	2022	2023	APC	95% CI	Period
Legal Amazon	6.89	6.3	6.15	6.42	6.37	5.31	5.96	6.24	6.71	6.33	5.74	-0.60	-1.9;0.80	2013-2023
Acre	5.89	6.07	5.53	5.57	5.13	4.60	5.59	4.71	5.35	5.59	5.11	-4.3*	-11.1;-1.9	2013-2018
												1.9	-0.8;8.8	2018-2023
Amazonas	5.35	5.41	4.97	5.55	7.09	4.95	5.09	5.28	5.62	4.95	4.52	-1.09	-3.5;1.4	2013-2023
Amapá	5.36	4.41	4.69	4.94	4.33	4.19	3.92	4.3	5.15	4.88	4.03	-0.9	-3.6;1.8	2013-2023
Pará	6.66	5.79	6.03	6.08	5.75	4.98	5.7	6.21	6.28	5.82	5.29	-0.7	-3.0;1.6	2013-2023
Rondônia	5.62	5.44	5.35	5.82	5.76	5.34	5.61	6.81	5.72	5.6	5.12	0.3	-1.4;1.9	2013-2023
Roraima	7.25	4.95	4.25	4.3	3.17	3.37	3.24	3.5	3.58	3.3	3.23	-14.8*	-22.7;-10.1	2013-2017
												0.3	-2.6;5.3	2017-2023
Tocantins	7.16	6.67	6.33	6.17	6.29	4.84	5.47	5.98	6.79	5.29	5.28	-2.1*	-3.8;-0.1	2013-2023
Maranhão **	8.75	7.93	7.8	6.34	7.97	6.93	7.55	7.97	9.26	10.01	8.89	-8.5*	-16.7;-3.9	2013-2016
												5.4*	3.8;7.4	2016;2023
Mato Grosso	5.46	5.74	5,00	4.89	4.71	4.13	4.91	5.77	5.86	4.89	5.03	-5.2*	-17.1;-1.1	2013-2018
												10.9*	0.25;16.6	2028-2021
												-11.1	-22.3;2.6	2021-2023

* Significantly different from zero at the alpha level = 0.05.
** Only municipalities in the Legal Amazon.

Table S3. Distribution of prevalence (%) and annual percentage change (APC) of overweight based on weight-for-height index (Z-score > +2) in children under 5 years old by state and sex. Legal Amazon, 2013-2023.

**Variables**	**2013**	**2014**	**2015**	**2016**	**2017**	**2018**	**2019**	**2020**	**2021**	**2022**	**2023**	**APC**	**95% CI**	**Period**
Legal Amazon	15.23	16.30	13.60	14.89	13.07	12.42	13.23	13.97	14.01	12.81	11.80	-4.7*	-6.6;-3.7	2013-2018
												4.0*	1.2;5.8	2018-2021
												-9.4*	-13.6;-5.1	2021-2023
Acre	15.93	16.86	13.66	16.75	13.24	13,00	13.84	14.54	15.82	14.03	12.88	-1.2	-2.8;0.1	2013-2023
Amazonas	13.94	15.81	13.23	14.3	12.33	11.28	12.31	13.64	13.05	12.74	11.35	-5.3*	-10;-3.5	2013-2018
												4.9	-1.5;8.2	2018-2021
												-8.3	-15.5;0.3	2021-2023
Amapá	12.71	17.89	13.52	14.1	12,00	12.76	12.86	14.06	13.21	12.68	12.13	-1.6*	-3.2;-0.20	2013-2023
Pará	14.51	13.65	12.23	14.39	12.79	12.37	12.98	13.56	13.83	12.68	11.74	-0.7	-2.1;0.5	2013-2023
Rondônia	14.02	14.14	14.35	12.94	13.63	13.3	13.85	12.5	12.88	11.43	10.43	-1.3*	-1.8;-0.3	2013-2021
												-10.0*	-13.7;-5.0	2021-2023
Roraima	17.76	18.63	11.48	11.89	10.74	10.21	10.99	10.79	10.99	9.38	8.62	-16.7*	-28.8;-7.9	2013-2016
												-2.18	-5.3;6.2	2016-2023
Tocantins	21.59	16.07	13.78	14.34	11.24	11.19	11.98	13.6	13.12	11.39	10.64	-13.1*	-21.7;-8.6	2013-2017
												4.3	-0.19;12.4	2017-2021
												-11.8*	-21.5;-2.9	2021-2023
Maranhão**	16.68	18.63	15.32	16.97	14.83	13.75	15.06	15.88	15.87	14.65	13.68	-4.7*	-8.3;-3.2	2013-2018
												4.2*	0.3;6.8	2018-2021
												-8.6*	-14.5;-2.7	2021-2023
Mato Grosso	15.99	17.96	14.92	13.42	12.56	11.94	11.75	12.46	13.17	11.44	12.85	-7.6*	-13.2;-5.1	2013-2018
												-1.2	-1.6;7.3	2018-2023
Male	2013	2014	2015	2016	2017	2018	2019	2020	2021	2022	2023	APC	95% CI	Period
Legal Amazon	16.13	16.96	14.29	15.57	13.77	13.1	13.91	14.75	14.69	13.54	12.48	-4.5*	-6.4;-3.5	2013-2018
												3.9*	1.2;5.7	2018-2021
												-9.1*	-13.2;-4.8	2021-2023
Acre	17.01	17.89	14.25	17.44	13.96	14.09	14.72	15.70	16.36	14.89	13.77	-4.4*	-5.8;-3.6	2013-2018
												5.3*	2.5;7.3	2018-2021
												-8.7*	-12.2;-4.4	2021-2023
Amazonas	14.95	16.92	13.85	14.99	13.08	12.02	13.01	14.54	13.76	13.61	12.19	-5.4*	-9.5;-3.7	2013-2018
												5.1	-1.3;8.1	2018-2021
												-7.8	-14.6;0.43	2021-2023
Amapá	13.62	18.8	14.28	14.37	12.66	13.69	13.82	14.63	14.11	12.89	12.65	-1.7*	-3.5;-0.03	2013-2023
Pará	15.44	14.48	12.89	15.25	13.55	13.07	13.79	14.44	14.52	13.51	12.45	-0.7	-2.3;0.7	2013-2023
Rondônia	14.58	15.03	15.55	13.73	14.34	13.99	14.64	13.25	13.72	12.14	11.04	-1.3	-2.1;2.9	2013-2021
												-9.9*	-16.1;-3.2	2021-2023
Roraima	18.73	19.93	12.68	12.33	11.17	10.8	11.93	11.64	11.83	10.18	9.48	-17.1*	-30.5;-6.7	2013-2016
												-1.7	-5.3;9.07	2016-2023
Tocantins	15.98	17.16	14.77	14.86	11.71	11.76	12.65	14.41	13.67	12.13	11.2	-7.5*	-10.6;-5.9	2013-2018
												6.7*	2.1;10.0	2018-2021
												-13.1*	-18.7;-6.8	2021-2023
Maranhão**	17.65	19.67	15.89	17.55	15.52	14.36	15.58	16.46	16.52	15.28	14.24	-4.9*	-8.5;-3.4	2013-2018
												4.2*	0.25;6.7	2018-2021
												-8.4*	-14.5;-2.6	2021-2023
Mato Grosso	16.74	18.55	15.4	13.89	13.17	12.36	12.18	13.04	13.8	11.95	11.45	-8.6*	-11.7;-6.2	2013-2018
												3.7	-1.5;6.7	2018-2021
												-8.8*	-14.7;-1.8	2021-2023
Female	2013	2014	2015	2016	2017	2018	2019	2020	2021	2022	2023	APC	95% CI	Period
Legal Amazon	14.53	15.21	12.91	14.19	12.34	11.72	12.51	13.15	13.29	12.04	11.09	-4.6*	-6.4;-3.7	2013-2018
												3.9*	1.2;5.7	2018-2021
												-9.6*	-13.5;-5.7	2021-2023
Acre	14.98	15.87	13.04	16.03	12.5	11.85	12.92	13.29	15.23	13.12	11.94	-1.4	-3.1;0.1	2013-2023
Amazonas	13.13	14.84	12.60	13.59	11.55	10.52	11.57	12.71	12.31	11.82	10.45	-0.59	-4.3;3.5	2013-2015
												-7.6*	-9.7;-5.5	2015-2018
												6.03*	3.5;8.5	2018-2021
												-9.7*	-14.4;-5.8	2021-2023
Amapá	11.96	17.03	12.74	13.84	11.32	11.81	11.85	13.45	12.25	12.46	11.6	-1.6*	-3.1;-0.4	2013-2023

Pará	13.73	12.9	11.59	13.5	11.99	11.64	12.14	12.64	13.1	11.81	10.99	-0.90	-2.2;0.4	2013-2023
Rondônia	13.58	13.39	13.2	12.13	12.87	12.57	13.04	11.69	11.97	10.67	9.78	-1.4*	-1.9;-0.8	2013-2021
												-9.9*	-12.5;-5.8	2021-2023
Roraima	17.01	17.58	10.36	11.44	10.29	9.59	9.97	9.90	10.08	8.53	7.74	-24.1*	-30.1;-11.2	2013-2015
												-3.5	-5.6;0.7	2015-2023
Tocantins	14.95	15.11	12.82	13.80	10.75	10.6	11.28	12.72	12.54	10.59	10.03	-7.4*	-10.5;-5.8	2013-2018
												6.3*	2.05;9.4	2018-2021
												-13.7*	-19.6;-7.2	2021-2023
Maranhão**	16.03	17.74	14.48	11.99	14.11	13.11	14.52	15.26	15.18	13.99	13.10	-1.1	-3.9;1.8	2013-2023
Mato Grosso	15.39	17.44	14.45	12.93	11.92	11.51	11.30	11.96	12.48	10.92	10.32	-8.6*	-17.1;-4.5	2013-2017
												-1.3	-3.9;6.1	2017-2023

* Significantly different from zero at the alpha level = 0.05.
** Only municipalities in the Legal Amazon.

Table S4. Distribution of prevalence (%) and annual percentage change (APC) of obesity based on weight-for-height index (Z-score > +3) in children under 5 years old by state and sex. Legal Amazon, 2013-2023.

**Variables**	**2013**	**2014**	**2015**	**2016**	**2017**	**2018**	**2019**	**2020**	**2021**	**2022**	**2023**	**APC**	**95% CI**	**Period**
Legal Amazon	9.04	9.72	7.15	8.47	6.62	5.77	6.63	6.76	7.2	6.19	5.42	-9.0*	-11.6;-7.7	2013-2018
												5.5*	1.2;8.9	2018-2021
												-13.4*	-19.2;-7.0	2021-2023
Acre	9.18	10.21	6.81	10.17	6.65	6.07	7.29	7.18	8.55	7.17	6.12	-2.7	-5.9;-0.2	2013-2023
Amazonas	7.89	9.52	6.93	8.15	6.39	5.03	6.18	6.38	6.28	5.87	4.85	-4.6*	-6.6;-2.7	2013-2023
Amapá	7.47	10.81	7.39	7.73	5.57	5.67	6.15	6.23	6.63	6.38	5.24	-11.6*	-24.4;-5.5	2013-2017
												0.1	-4.3;13.2	2017-2023
Pará	8.24	7.57	6.08	7.72	6.16	5.7	6.35	6.54	7.01	5.98	5.32	-2.4	-5.3;0.5	2013-2023
Rondônia	7.89	8.13	7.54	6.91	6.54	6.15	7.01	5.5	6.29	5.23	4.58	-4.7*	-6.2;-3.3	2013-2023
Roraima	9.36	10.37	5.31	6.38	4.72	4.59	4.9	4.21	4.49	3.67	3.36	-20.5*	-29.3;-15.2	2013-2016
												-5.5*	-7.7;-2.3	2016-2023
Tocantins	9.21	9.48	7.17	8.06	5.37	4.85	6.02	6.72	6.74	5.01	4.52	-12.5*	-19.1;-9.3	2013-2018
												10.3*	1.8;16.7	2018-2021
												-22.5*	-32.4;-10.9	2021-2023
Maranhão**	10.76	12.34	8.51	10.35	8.13	6.75	7.89	8.14	8.84	7.9	7.1	-8.6*	-20.6;-4.1	2013-2018
												1.4	-4.2;16.1	2018-2023
Mato Grosso	9.45	11.45	7.92	7.38	6.19	5.44	5.52	5.91	6.6	5.18	4.84	-13.9*	-17.0;-12.3	2013-2018
												5.6*	0.01;9.7	2018-2021
												-14.1*	-20.8;-7.2	2021-2023
Male	2013	2014	2015	2016	2017	2018	2019	2020	2021	2022	2023	APC	95% CI	Period
Legal Amazon	9.65	10.28	7.5	8.9	7.08	6.19	7.14	7.28	7.69	6.7	5.83	-8.7*	-10.4;-7.7	2013-2018
												5.7*	1.8;8.6	2018-2021
												-13.2*	-17.8;-8.1	2021-2023
Acre	9.94	10.91	7.08	10.43	7.21	6.65	7.84	7.78	9.12	7.82	6.65	-2.4*	-5.0;-0.2	2013-2023
Amazonas	8.56	10.1	7.17	8.49	6.84	5.46	6.67	6.92	6.78	6.47	5.33	-4.1*	-6.5;-2.0	2013-2023
Amapá	7.77	11.28	7.68	8.02	5.88	6.35	6.76	6.68	7.32	6.4	5.55	-4.0*	-6.9;-1.3	2013-2023
Pará	8.82	8.05	6.38	8.29	6.68	6.13	6.9	7.06	7.74	6.55	5.78	-2.0	-5.5;1.4	2013-2023
Rondônia	8.3	8.75	8.24	7.35	7.01	6.47	7.53	5.9	6.63	5.62	4.89	-4.7*	-6.5;-3.1	2013-2023
Roraima	9.85	11.27	5.74	6.39	4.86	5.06	5.46	4.77	4.78	3.93	3.75	-20.9*	-32.7;-12.4	2013-2016
												-4.8	-7.9;2.2	2016-2023
Tocantins	9.38	9.99	7.68	8.4	5.62	5.17	6.53	7.27	7.17	5.45	4.63	-12.1*	-19.8;-8.6	2013-2018
												11.0*	1.8;17.9	2018-2021
												-23.9*	-34.3;-11.7	2021-2023
Maranhão**	11.55	13.11	9.00	10.79	8.65	7.21	8.44	8.72	9.46	8.5	7.54	-9.7*	-12.5;-8.3	2013-2018
												6.9*	1.8;10.6	2018-2021
												-10.2*	-16.6;-3.0	2021-2023
Mato Grosso	10.08	11.73	8.04	7.72	6.54	5.66	5.76	6.2	6.9	5.46	5.12	-13.7*	-15.8;-12.5	2013-2018
												5.7*	1.1;8.9	2018-2021
												-13.7*	-18.8;-8.4	2021-2023
Female	2013	2014	2015	2016	2017	2018	2019	2020	2021	2022	2023	APC	95% CI	Period
Legal Amazon	8.55	9.23	6.81	8.02	6.14	5.33	6.1	6.21	6.69	5.64	4.99	-9.6*	-11.5;-8.6	2013-2018
												5.4*	1.2;8.5	2018-2021
												-13.6*	-18.6;-7.9	2021-2023
Acre	8.51	9.53	6.53	9.89	6.07	5.45	6.71	6.53	7.94	6.48	5.56	-3.2*	-6.3;-0.3	2013-2023
Amazonas	7.35	9.01	6.68	7.8	5.93	4.58	5.67	5.82	5.76	5.24	4.33	-5.2*	-7.4;-3.3	2013-2023
Amapá	7.23	10.36	7.09	7.44	5.26	4.98	5.51	5.74	5.9	6.36	4.92	-11.4*	-20.6;-7.7	2013-2018
												2.9	-2.3;13.7	2018-2023
Pará	7.76	7.13	5.79	7.13	5.61	5.26	5.78	5.99	6.53	5.38	4.83	-2.7*	-4.8;-0.8	2013-2023
Rondônia	7.57	7.61	6.87	6.46	6.04	5.81	6.47	5.07	5.92	4.82	4.25	-4.8*	-6.3;-3.3	2013-2023
Roraima	8.99	9.64	4.9	6.38	4.57	4.09	4.29	3.62	4.17	3.39	2.96	-27.5*	-33.7;-14.8	2013-2015
												-7.3*	-9.5;-2.4	2015-2023
Tocantins	9.07	9.03	6.68	7.71	5.11	4.52	5.5	6.13	6.28	4.53	4.4	-13.2*	-21.1;-9.6	2013-2018
												9.5*	0.3;16.4	2018-2021
												-20.8*	-31.9;-8.5	2021-2023
Maranhão**	10.13	11.68	8.04	9.89	7.59	6.27	7.31	7.53	8.19	7.27	6.65	-9.0*	-20.1;-4.9	2013-2018
												1.3	-3.9;15.2	2018-2023
Mato Grosso	8.94	11.2	7.81	7.03	5.83	5.22	5.27	5.61	6.27	4.9	4.54	-14.2*	-18.4;-12.1	2013-2018
												5.5	-1.4;10.4	2018-2021
												-14.6*	-22.5;-4.9	2021-2023

* Significantly different from zero at the alpha level = 0.05.
** Only municipalities in the Legal Amazon.

Table S5. Distribution of prevalence (%) and annual percentage change (APC) of underweight (BMI < 18.5 kg/m²) in adults by state and sex. Legal Amazon, 2013-2023.

Variables	2013	2014	2015	2016	2017	2018	2019	2020	2021	2022	2023	APC	95% CI	Period
Legal Amazon	3.87	3.66	2.86	2.99	2.64	2.48	2.63	2.23	2.17	2.27	2.26	-13.5*	-15.8;-9.4	2013-2015
												-5.2*	-6.7;-3.9	2015-2021
												2.2	-2.9;5.2	2021-2023
Acre	3.32	3.94	2.57	2.87	2.39	2.18	2.42	2.06	2.1	2.22	2.13	-9.7*	-15.3;-7.4	2013-2018
												-0.7	-3.8;5.4	2018-2023
Amazonas	2.79	3.16	2.16	2.46	2.04	2.01	2.3	1.85	1.87	1.99	2.07	-9.0*	-15.8;-6.0	2013-2017
												-1.1	-3.1;3.2	2017-2023
Amapá	2.72	2.48	2.2	2.58	2.32	2.24	2.37	1.81	1.79	1.79	1.81	-4.2*	-6.1;-2.3	2013-2023
Pará	3.69	3.52	2.99	3.08	2.71	2.53	2.62	2.22	2.17	2.24	2.2	-6.3*	-7.5;-5.8	2013-2021
												2.2	-4.0;5.5	2021-2023
Rondônia	4.22	4.2	3.13	3.03	2.84	2.75	2.78	2.36	2.26	2.56	2.56	-15.5*	-19.2;-8.6	2013-2015
												-5.5*	-8.1;-2.6	2015-2021
												6.6	-1.8;11.7	2021-2023
Roraima	9.57	8.54	2.02	2.55	1.93	1.99	2.21	1.65	1.82	1.84	2.05	-59.3*	-65.7;-40.7	2013-2015
												-3.7	-8.2;2.9	2015-2023
Tocantins	3.97	3.81	3.25	3.04	2.72	2.54	2.66	2.21	2.37	2.4	2.33	-9.5*	-12.7;-7.1	2013-2017
												-5.0*	-9.8;-1.9	2017-2020
												0.3	-2.3;4.2	2020-2023
Maranhão**	4.16	3.8	3.06	3.33	3.06	2.82	3.1	2.73	2.59	2.72	2.64	-12.5*	-15.8;-5.7	2013-2015
												-2.8*	-3.8;-0.5	2015-2023
Mato Grosso	3.28	3.22	2.67	2.55	2.18	2.06	2.11	1.88	1.87	1.98	2.05	-10.4*	-15.9;-7.3	2013-2017
												4.3*	-9.9;-1.5	2017-2021
												5.7	-1.5;10.6	2021-2023
Male	2013	2014	2015	2016	2017	2018	2019	2020	2021	2022	2023	APC	95% CI	Period
Legal Amazon	3.77	3.65	2.09	2.05	1.85	1.77	1.76	1.74	1.76	1.96	2.01	-22.8*	-29.8;-19.1	2013-2016
												-2.4	-9.7;2.0	2016-2020
												5.9*	1.5;13.4	2020-2023
Acre	3.63	8.67	1.39	1.77	1.58	1.71	1.53	1.7	1.59	1.61	1.62	-38.4*	-56.9;-24.3	2013-2016
												1.1	-5.0;11.0	2016-2023
Amazonas	2.25	2.6	1.55	1.58	1.47	1.43	1.5	1.42	1.52	1.66	1.8	-17.0*	-24.1;-13.2	2013-2016
												-0.9	-7.4;3.4	2016-2020
												7.5*	3.0;15.9	2020-2023
Amapá	7.14	2.91	1.81	1.66	1.57	1.62	1.55	1.43	1.36	1.46	1.5	-48.7*	-52.9;-40.6	2013-2015
												-2.4*	-4.5;-0.0	2015-2023
Pará	3.23	2.99	2.3	2.15	1.75	1.69	1.68	1.69	1.71	1.87	1.84	-15.0*	-17.9;-13.6	2013-2017
												-1.8	-13.0;0.3	2017-2020
												4.5*	1.7;8.8	2020-2023
Rondônia	3.3	4.81	2.13	2.19	1.97	2.03	1.94	2.2	2.05	2.39	2.38	-24.4*	-37.3;-16.7	2013-2016
												2.4	-0.8;6.4	2016-2023
Roraima	4.17	5.61	1.32	1.93	1.54	1.15	1.42	1.53	1.64	1.71	2.01	-45.3*	-52.7;-33.0	2013-2015
												-12.7	-18.3;8.7	2015-2018
												8.8	-0.8;25.4	2018-2023
Tocantins	4.78	4.02	2.51	2.11	2.06	1.93	1.9	1.97	2.08	2.32	2.27	-25.4*	-33.0;-21.4	2013-2016
												-2.7	-12.4;5.8	2016-2019
												5.5*	2.0;12.7	2019-2023
Maranhão**	3.67	4.06	2.21	2.33	2.27	2.17	2.09	2.12	2.05	2.29	2.36	-20.0*	-33.7;-9.8	2013-2016
												0.5	-3.2;8.4	2016-2023
Mato Grosso	4.53	3.45	2.07	1.99	1.79	1.58	1.55	1.72	1.68	1.87	2.01	-32.4*	-35.3;-29.0	2013-2015
												-10.4*	-12.7;-8.0	2015-2018
												2.8*	0.1;5.4	2018-2021
												8.7*	2.6-13.9	2021-2023
Female	2013	2014	2015	2016	2017	2018	2019	2020	2021	2022	2023	APC	95% CI	Period
Legal Amazon	3.87	3.66	2.92	3.09	2.71	2.55	2.74	2.36	2.29	2.38	2.35	-12.4*	-14.7;-8.7	2013-2015
												-4.6*	-5.9;-3.5	2015-2021
												1.4	-2.9;4.3	2021-2023
Acre	3.31	3.88	2.64	2.96	2.44	2.21	2.5	2.12	2.19	2.38	2.29	-9.3*	-15.5;-6.7	2013-2018
												0.2	-3.2;7.3	2018-2023
Amazonas	2.8	3.17	2.22	2.57	2.09	2.08	2.42	1.99	2	2.12	2.19	-8.2*	-15.7;-4.7	2013-2017
												-0.6	-2.9;5.5	2017-2023
Amapá	2.71	2.48	2.21	2.63	2.35	2.27	2.42	1.9	1.92	1.88	1.9	-3.5*	-5.1;-2.0	2013-2023
Pará	3.7	3.52	3.03	3.16	2.77	2.59	2.71	2.35	2.28	2.35	2.32	-5.7*	-7.1;-5.1	2013-2021
												2.1	-3.8;5.3	2021-2023
Rondônia	4.23	4.2	3.29	3.24	3.01	2.88	2.95	2.4	2.33	2.62	2.64	-12.2*	-15.8;-6.3	2013-2015

												-5.9*	-8.6;-3.2	2015-2021
												6.4	-1.6;11.2	2021-2023
Roraima	9.6	8.55	2.05	2.59	1.96	2.03	2.28	1.69	1.89	1.88	2.07	-58.9*	-64.7;-45.1	2013-2015
												-3.5	-7.3;1.7	2015-2023
Tocantins	3.95	3.81	3.33	3.23	2.84	2.66	2.8	2.29	2.46	2.44	2.36	-6.9*	-9.2;-5.9	2013-2020
												0.3	-4.0;6.3	2020-2023
Maranhão**	4.16	3.79	3.11	3.4	3.11	2.86	3.2	2.86	2.73	2.84	2.73	-11.8*	-14.8;-5.4	2013-2015
												-2.3*	-3.2;-0.5	2015-2023
Mato Grosso	3.24	3.22	2.73	2.65	2.24	2.15	2.22	1.93	1.94	2.03	2.06	-9.3*	-13.6;-6.7	2013-2017
												-4.4*	-9.0;-2.2	2017-2021
												4.3	-1.8;8.3	2021-2023

* Significantly different from zero at the alpha level = 0.05.
** Only municipalities in the Legal Amazon.

Table S6. Distribution of prevalence (%) and annual percentage change (APC) of overweight (BMI ≥ 25.0 kg/m²) in adults by state and sex. Legal Amazon, 2013-2023.

**Variables**	**2013**	**2014**	**2015**	**2016**	**2017**	**2018**	**2019**	**2020**	**2021**	**2022**	**2023**	**APC**	**95% CI**	**Period**
Legal Amazon	48.85	51.10	55.04	55.00	56.33	58.69	59.75	62.88	63.87	63.56	64.64	5.1*	3.2;7.1	2013-2015
												2.8*	2.1;3.5	2015-2021
												0.4	-1.5;2.3	2021-2023
Acre	53.00	54.00	57.00	57.5	58.65	60.03	59.9	62.16	62.36	62.78	63.63	3.2*	2.7;4.3	2013-2016
												1.4*	1.2;1.6	2016-2023
Amazonas	56.00	56.00	61.00	59.64	60.13	63.17	63.82	67.52	67.97	67.17	67.45	2.2*	1.6;2.7	2013-2023
Amapá	60.00	62.00	64.00	60.73	62.11	63.81	63.55	67.76	68.39	66.58	68.38	1.3*	0.6;1.9	2013-2023
Pará	47.00	50.00	53.00	53.1	54.84	57.55	58.47	62.02	62.71	62.37	63.67	3.6*	3.2;4.6	2013-2020
												1.1	-1.7;2.5	2020-2023
Rondônia	49.00	50.00	57.00	58.12	59.14	60.75	61.57	64.45	65.85	64.35	64.75	8.7*	6.2;10.5	2013-2015
												2.6*	2.1;3.2	2015-2021
												1.0	-2.7;1.0	2021-2023
Roraima	49.00	50.00	63.00	61.61	64.37	64.13	64.47	68.98	69.53	68.00	66.83	15.4*	8.7;20.6	2013-2015
												1.6*	0.5;2.2	2015-2023
Tocantins	52.00	53.00	56.00	57.31	57.82	59.1	60.64	63.24	64.21	62.39	63.38	2.6*	2.3;3.0	2013-2021
												-1.3	-2.8;09	2021-2023
Maranhão**	45.00	48.00	51.00	49.95	51.04	53.84	54.66	56.53	58.35	58.41	60.29	2.7*	2.3;3.1	2013-2023
Mato Grosso	56.00	58.00	61.00	61.58	64.17	65.09	66.86	67.94	69.37	68.38	69.10	4.1*	3.7;4.5	2013-2015
												2.6*	2.4;2.8	2015-2018
												1.9*	1.6;2.1	2018-2021
												-0.5	-1.1;0.0	2021-2023
Male	2013	2014	2015	2016	2017	2018	2019	2020	2021	2022	2023	APC	95% CI	Period
Legal Amazon	49.1	50.1	57.51	57.71	58.16	58.85	58.33	60.62	61.53	60.42	60.68	9.5*	5.8;12.1	2013-2015
												1.0*	0.4;1.3	2015-2023
Acre	41.00	32.00	52.00	51.42	48.96	48.78	46.4	51.19	51.16	53.90	55.21	3.2*	1.4;5.1	2013-2023
Amazonas	51.00	51.00	66.00	64.63	63.72	62.74	62.87	65.74	65.88	64.33	63.29	15.4*	7.5;22.1	2013-2015
												0.2	-1.1;0.9	2015-2023
Amapá	58.00	73.00	62.00	59.59	61.24	63.51	58.94	65.52	66.50	63.14	64.76	0.1	-1.1;1.3	2013-2023
Pará	47.00	49.00	55.00	55.8	55.97	57.17	57.05	59.16	60.34	59.27	60.04	9.1*	5.8;11.5	2013-2015
												1.3*	0.8;1.6	2015-2023
Rondônia	52.00	50.00	60.00	57.54	58.64	59.75	59.63	61.10	62.69	60.78	60.66	8.6*	3.8;12.6	2013-2015
												0.9	-0.2;1.4	2015-2023
Roraima	61.00	57.00	69.00	62.45	63.77	62.63	60.73	66.17	65.62	63.60	60.62	0.4	-0.4;1.1	2013-2023
Tocantins	52.00	53.00	56.00	57.34	56.68	58.06	58.32	59.44	60.97	58.78	59.54	4.2*	2.8;5.4	2013-2015
												1.2*	0.9;1.6	2015-2021
												-1.0	-2.3;0.3	2021-2023
Maranhão**	43.00	52.00	52.00	51.29	51.14	52.54	51.87	53.57	54.70	54.64	55.58	1.4*	0.6;2.2	2013-2023
Mato Grosso	55.00	57.00	61.00	61.34	64.18	64.43	64.74	65.14	66.95	64.70	65.02	5.4*	4.3;6.7	2013-2015
												2.2*	1.5;2.8	2015-2018
												0.8*	0.1;1.4	2018-2021
												-1.1*	-2.5;-0.0	2021-2023
Female	2013	2014	2015	2016	2017	2018	2019	2020	2021	2022	2023	APC	95% CI	Period
Legal Amazon	48.84	51.11	54.87	54.70	56.17	58.68	59.93	63.49	64.56	64.68	66.17	3.1*	2.7;3.3	2013-2023
Acre	53.00	54.00	58.00	58.02	59.23	60.69	61.05	64.23	64.37	64.98	66.27	4.5*	2.9;5.9	2013-2015
												1.9*	1.5;2.1	2015-2023
Amazonas	56.00	56.00	61.00	59.00	59.76	63.21	63.97	68.11	68.76	68.4	69.26	2.4*	1.8;3.0	2013-2023
Amapá	60.00	62.00	64.00	60.79	62.15	63.83	63.82	68.33	68.92	67.56	69.48	1.4*	0.8;2.0	2013-2023
Pará	47.00	50.00	53.00	52.88	54.78	57.57	58.60	62.71	63.3	63.29	64.92	3.2*	2.7;3.7	2013-2023
Rondônia	49.00	50.00	56.00	58.26	59.22	60.94	61.96	65.46	66.77	65.75	66.53	6.8*	4.4;12.7	2013-2016
												2.2*	0.6;2.8	2016-2023
Roraima	49.00	50.00	63.00	61.56	64.41	64.21	64.82	69.96	70.97	69.80	69.41	14.8*	7.5;21.1	2013-2015
												2.1*	0.7;2.7	2015-2023
Tocantins	52.00	53.00	56.00	57.32	58.01	59.30	61.13	64.36	65.25	64.10	65.32	2.8*	2.6;3.4	2013-2021
												-0.4	-2.0;1.8	2021-2023
Maranhão**	45.00	48.00	51.00	49.86	51.04	53.94	54.94	57.16	59.25	59.46	61.70	3.0*	2.4;3.5	2013-2023
Mato Grosso	56.00	58.00	61.00	61.62	64.17	65.21	67.28	68.86	70.21	69.99	71.05	4.0*	3.5;4.4	2013-2015
												2.7*	2.5;2.8	2015-2020
												0.9*	0.6;1.2	2020-2023

* Significantly different from zero at the alpha level = 0.05.
** Only municipalities in the Legal Amazon.

Table S7. Distribution of prevalence (%) and annual percentage change (APC) of obesity (BMI ≥ 30.0 kg/m²) in adults by state and sex. Legal Amazon, 2013-2023.

Variables	2013	2014	2015	2016	2017	2018	2019	2020	2021	2022	2023	APC	95% CI	Period
Legal Amazon	17.10	18.4	20.67	20.88	21.61	23.06	24.05	26.27	27.32	27.34	28.49	9.2*	7.0;11.9	2013-2015
												3.9*	2.7;5.2	2015-2018
												6.5*	5.2;7.7	2018-2021
												1.1	-1.3;3.1	2021-2023
Acre	19.85	20.2	22.36	22.67	23.02	24.45	24.59	25.97	27.31	27.51	28.22	3.7*	3.3;4.0	2013-2023
Amazonas	20.77	21.33	24.52	23.97	24.45	26.13	26.93	30.17	30.96	30.38	30.91	8.0*	5.5;11.0	2013-2015
												2.6*	1.1;4.0	2015-2018
												7.0*	5.5;8.5	2018-2021
												-1.6	-4.5;0.8	2021-2023
Amapá	24.16	25.69	27.18	24.87	24.78	26.05	26.47	29.26	30.51	29.62	31.99	2.5*	1.0;3.9	2013-2023
Pará	15.35	16.78	18.99	19.03	19.68	21.24	22.29	24.5	25.37	25.41	26.62	9.5*	4.3;17.3	2013-2015
												5.0	-2.4;7.4	2015-2023
Rondônia	19.47	20.77	23.89	24.79	25.63	26.94	27.51	29.68	31.62	30.09	30.55	10.9*	7.3;14.3	2013-2015
												4.7*	3.9;5.3	2015-2021
												-1.1	-4.2;2.3	2021-2023
Roraima	21.49	21.66	26.98	25.47	27.91	27.53	27.69	31.17	32.23	31.10	30.62	12.0*	4.9;19.9	2013-2015
												3.0	-3.1;3.7	2015-2023
Tocantins	20.19	21.47	23.07	23.66	23.33	24.3	25.67	28.1	28.42	27.24	28.37	3.4*	2.4;4.5	2013-2023
Maranhão **	14.01	15.55	16.79	16.35	17.09	18.56	19.11	20.24	21.32	21.52	23.23	4.7*	4.0;5.4	2013-2023
Mato Grosso	24.10	25.65	27.53	27.92	29.91	30.89	32.67	33.69	35.59	33.99	34.8	4.7*	4.4;5.0	2013-2021
Male	2013	2014	2015	2016	2017	2018	2019	2020	2021	2022	2023	APC	95% CI	Period
Legal Amazon	14.47	14.64	19.66	19.73	20,00	20.29	19.9	21.78	23.08	22.41	23.02	18.3*	8.3;28.1	2013-2015
												2.6*	0.5;3.6	2015-2023
Acre	11.40	6.74	15.72	16.51	13.57	15.35	14.1	15.82	17.52	18.3	19.24	6.7*	3.5;9.8	2013-2023
Amazonas	15.65	15.98	26.63	24.78	23.98	23.4	23.28	26.25	27.02	27.75	25.36	29.9*	12.5;47.2	2013-2015
												1.5	-1.7;2.9	2015-2023
Amapá	26.19	31.07	22.71	19.81	20.72	17.95	20.73	24.12	26.08	24.21	26.59	-11.1*	-25.9;-3.7	2013-2017
												6.5*	2.0;18.1	2017-2023
Pará	12.36	12.69	16.98	17.68	17.62	22.01	18.27	19.81	20.89	20.49	21.48	16.1*	11.5;25.3	2013-2016
												2.2*	0.6;3.4	2016-2023
Rondônia	16.65	15.80	22.76	20.74	21.55	23.26	21.6	23.58	25.87	24.23	24.14	17.7*	5.8;30.9	2013-2015
												2.4	-4.4;3.5	2015-2023
Roraima	22.91	24.30	29.81	22.10	24.42	19.74	21.40	25.55	26.32	24.19	23.55	10.2*	4.1;17.5	2013-2015
												-9.9*	-12.8;-6.8	2015-2018
												9.8*	6.1;13.7	2018-2021
												-7.4*	-14.6;-1.3	2021-2023
Tocantins	16.48	16.1	18.87	19.75	19.11	19.74	20.25	21.88	23.41	21.56	22.31	3.4*	1.7;4.9	2013-2023
Maranhão**	10.57	12.46	14.07	13.68	14.09	14.35	13.72	14.87	15.79	16.03	17.31	14.2*	9.6;17.6	2013-2015
												0.1	-1.8;1.3	2015-2019
												5.1*	3.7;8.0	2019-2023
Mato Grosso	19.15	19.83	23.05	23.7	25.42	25.80	26.39	27.32	30.7	27.91	28.46	9.4*	5.8;16.8	2013-2016
												2.8*	0.3;3.7	2016-2023
Amazônia Legal	14.47	14.64	19.66	19.73	20,00	20.29	19.90	21.78	23.08	22.41	23.02	18.3*	8.3;28.1	2013-2015
												2.6*	0.5;3.6	2015-2023
Female	2013	2014	2015	2016	2017	2018	2019	2020	2021	2022	2023	APC	95% CI	Period
Legal Amazon	17.13	18.44	20.74	21,00	21.76	23.33	24.58	27.48	28.57	29.09	30.61	5.9*	5.3;6.6	2013-2023
Acre	19.9	20.35	22.73	23.19	23.59	24.98	25.49	27.88	29.07	29.8	31.04	4.5*	4.2;4.9	2013-2023
Amazonas	20.8	21.38	24.33	23.86	24.49	26.42	27.46	31.47	32.47	32.36	33.32	5.3*	4.4;6.1	2013-2023
Amapá	24.16	25.66	27.39	25.13	24.93	26.21	26.81	30.56	31.79	31.16	33.63	-0.2	-5.7;2.3	2013-2017
												4.9*	3.4;9.2	2017-2023
Pará	15.39	16.83	19.1	19.14	19.82	21.46	22.66	25.63	26.48	26.88	28.38	6.2*	5.4;7.0	2013-2023
Rondônia	19.49	20.80	24.08	25.77	26.41	27.87	28.69	31.52	33.3	32.39	33.33	12.0*	8.7;14.8	2013-2015
												5.4*	4.6;6.2	2015-2021
												0.4	-2.2;3.2	2021-2023
Roraima	21.49	21.65	26.86	25.68	28.11	27.74	28.28	33.12	34.4	33.63	33.55	5.0*	3.7;6.2	2013-2023
Tocantins	20.28	21.58	23.53	24.50	24.08	25.16	26.81	29.94	30.04	29.92	31.44	4.4*	3.3;5.5	2013-2023
Maranhão**	14.04	15.56	16.94	16.54	17.26	18.83	19.68	21.42	22.73	23.15	25.10	5.6*	4.6;6.6	2013-2023
Mato Grosso	24.28	25.78	27.97	28.74	30.65	31.81	33.91	35.8	37.29	36.65	37.79	5.6*	5.2;6.2	2013-2020
												1.6	-1.7;3.1	2020-2023

* Significantly different from zero at the alpha level = 0.05.
** Only municipalities in the Legal Amazon.

Table S8. Distribution of prevalence (%) and annual percentage change (APC) of underweight (BMI < 22.0 kg/m²) in older adults by state and sex. Legal Amazon, 2013-2023.

Variables	2013	2014	2015	2016	2017	2018	2019	2020	2021	2022	2023	APC	95% CI	Period
Legal Amazon	17.47	17.93	14.68	14.63	13.93	13.05	12.95	12.95	12.69	12.90	13.04	-6.3*	-8.3;-5.2	2013-2018
												-0.1	-1.5;2.3	2018-2023
Acre	23.47	20.51	17.52	16.11	16.24	14.8	15.6	14.6	15.39	14.89	14.72	-14.8*	-15.9;-13.6	2013-2015
												-4.3*	-4.9;-3.1	2015-2018
												-0.4	-0.9;0.1	2018-2023
Amazonas	21.02	17.20	12.07	12.62	11.58	11.01	10.95	10.78	10.48	10.76	11.32	-23.5*	-24.8;-22.2	2013-2015
												-4.2*	-5.2;-3.2	2015-2018
												-2.0*	-3.0;-0.9	2018-2021
												3.3*	1.0;5.4	2021-2023
Amapá	6.67	12.10	13.7	15.14	14.06	11.77	11.56	10.73	10.53	10.61	10.56	40.5*	13.9;65.3	2013-2015
												-5.2*	-8.9;-3.4	2015-2023
Pará	15.91	17.32	14.42	14.47	13.96	13.05	12.82	12.91	12.69	12.95	12.96	-5.2*	-7.2;-4.0	2013-2018
												-0.1	-0.2;1.6	2018-2023
Rondônia	14.96	10.77	12.01	12.95	11.82	11.50	11.59	11.70	11.5	12.14	12.50	-0.5	-2.0;1.0	2013-2023
Roraima	18.75	21.09	10.28	10.98	10.16	11.18	10.91	10.73	10.24	11.52	11.50	-22.2*	-34.4;-14.7	2013-2016
												1.7	-1.1;5.4	2016-2023
Tocantins	17.82	18.06	16.28	15.67	15.74	14.68	14.46	14.75	14.14	14.90	14.89	-3.8*	-4.7;-3.2	2013-2019
												0.98	-0.98;0.33	2019-2023
Maranhão**	22.44	19.69	17.27	17.52	17.35	16.09	15.93	15.67	15.37	14.90	14.85	-11.2*	-12.88;-8.26	2013-2015
												-2.24*	-2.6;-1.8	2015-2023
Mato Grosso	16.11	16.66	13.46	12.77	11.55	10.98	10.69	11.51	10.97	11.86	12.21	-9.0*	-10.8;-7.8	2013-2018
												2.5*	1.0;5.0	2018-2023
Male	2013	2014	2015	2016	2017	2018	2019	2020	2021	2022	2023	APC	95% CI	Period
Legal Amazon	19.30	19.32	14.98	15.12	14.53	13.73	13.63	13.45	13.15	13.45	13.76	-10.8*	-16.8;-6.8	2013-2016
												-1.3	-2.5;0.8	2016-2023
Acre	27.32	24.71	17.15	15.82	16.46	15.31	17.89	15.33	15.79	15.72	15.70	-24.8*	-29,0;-16.2	2013-2015
												-1.0	-2.3;0.6	2015-2023
Amazonas	21.74	22.09	11.16	11.89	11.13	10.9	10.88	10.9	10.69	10.77	11.43	-23.3*	-34.1;-17.4	2013-2016
												0.2	-1.9;3.3	2016-2023
Amapá	11.11	11.11	14.07	16.24	14.02	13.33	11,00	10.77	10.85	10.67	10.76	15.2*	10.9;20.5	2013-2016
												-11.4*	-14.2;-7.2	2016-2019
												-1.2	-3.7;5.3	2019-2023
Pará	17.21	15.42	14.06	14.15	14.02	13.33	12.91	13.09	12.94	13.16	13.30	-9.8*	-12.8;-3.5	2013-2015
												-1.0	-1.9;1.7	2015-2023
Rondônia	18.37	15.22	13.7	15.31	13.58	13.10	13.57	13.61	13.4	14.04	14.58	-12.2*	-17.9;-1.0	2013-2015
												0.1	-2.4;7.0	2015-2023
Roraima	11.76	-	12.17	12.17	11.13	13.52	12.01	11.79	11.05	12.21	13.02	0.1	-1.5;1.7	2013-2023
Tocantins	20.16	22.05	17.90	17.1	17.34	16.16	15.58	16.11	15.04	16.19	16.20	-5.8*	-9.6;-4.1	2013-2018
												0.1	-1.8;4.0	2018-2023
Maranhão**	25.23	22.00	17.51	17.69	17.20	15.83	16.2	15.81	15.44	14.82	15.02	-17.0*	-18.6;-14.4	2013-2015
												-2.4*	-2.9;-1.9	2015-2023
Mato Grosso	17.94	19.03	15.77	14.54	13.37	12.74	12.51	13.46	12.72	13.47	13.84	-8.3*	-10.0;-6.9	2013-2018
												1.9*	0.3;4.0	2018-2023
Female	2013	2014	2015	2016	2017	2018	2019	2020	2021	2022	2023	APC	95% CI	Period
Legal Amazon	16.26	17.72	14.52	14.31	13.55	12.62	12.52	12.55	12.34	12.45	12.47	-6.3*	-7.7;-5.3	2013-2018
												-0.3	-1.5;1.5	2018-2023
Acre	19.79	19.85	17.68	16.26	16.13	14.54	14.27	14.04	15.07	14.25	13.94	-6.5*	-8.2;-5.3	2013-2018
												-0.5	-1.8;1.3	2018-2023
Amazonas	20.58	16.74	12.52	13.06	11.84	11.08	10.99	10.69	10.32	10.76	11.24	-21.2*	-22.8;-19.6	2013-2015
												-3.9*	-4.7;-3.3	2015-2021
												5.2*	1.2;7.6	2021-2023
Amapá	4.76	12.16	13.52	14.44	14.08	10.9	11.91	10.70	10.26	10.56	10.4	55.6*	19.6;90.2	2013-2015
												-5.3*	-9.5;-3.0	2015-2023
Pará	15.06	17.62	14.62	14.65	13.92	12.88	12.76	12.76	12.49	12.79	12.69	-4.6*	-6.4;-3.8	2013-2019
												0.3	-1.8;4.0	2019-2023
Rondônia	12.82	10.37	10.75	11.07	10.51	10.34	10.2	10.06	9.95	10.58	10.76	-1.0	-2.1;0.0	2013-2023
Roraima	22.58	21.11	9.66	10.33	9.59	9.79	10.22	9.83	9.58	10.94	10.20	-39.9*	-44.9;-33.6	2013-2015
												-0.1	-1.5;1.7	2015-2023
Tocantins	16.27	16.72	15.24	14.58	14.55	13.55	13.62	13.57	13.35	13.74	13.69	-4.1*	-5.7;-3.2	2013-2018
												-0.1	-1.1;1.9	2018-2023
Maranhão**	21,00	19.61	17.16	17.43	17.44	16.24	15.77	15.57	15.32	14.96	14.73	-8.7*	-10.3;-5.7	2013-2015
												-2.3*	-2.7;-1.8	2015-2023
Mato Grosso	14.8	15.82	12.02	11.47	10.28	9.77	9.47	9.93	9.57	10.55	10.87	-10.1*	-12.79;-8.3	2013-2018
												2.6*	0.3;6.0	2018-2023

* Significantly different from zero at the alpha level = 0.05.

** Only municipalities in the Legal Amazon.

Table S9. Distribution of prevalence (%) and annual percentage change (APC) of overweight (BMI ≥ 27.0 kg/m²) in older adults by state and sex. Legal Amazon, 2013-2023.

**Variables**	**2013**	**2014**	**2015**	**2016**	**2017**	**2018**	**2019**	**2020**	**2021**	**2022**	**2023**	**APC**	**95%CI**	**Period**
Legal Amazon	42.35	41.96	45.78	45.84	46.92	48.31	48.23	47.48	47.75	47.97	48.22	3.4*	2.5;5.1	2013-2017
												0.1	-0.7;0.6	2017-2023
Acre	37.33	40.14	41.86	45.45	45.30	45.74	45.99	44.94	45.14	47.79	47.18	6.3*	4.5;9.6	2013-2016
												0.6*	0.1;1.1	2016-2023
Amazonas	46.67	58.79	49.66	46.87	46.91	50.20	47.60	52.60	51.47	50.43	51.74	0.1	-1.1;1.2	2013-2016
Amapá	37.37	42.42	51.36	50.41	51.61	52.93	53.44	53.18	53.52	53.06	52.08	16.6*	15.3;18.1	2013-2015
												1.6*	1.1;2.4	2015-2019
												-0.4*	-1.6;0.1	2019-2023
Pará	42.64	42.35	45.27	45.47	46.48	47.95	47.95	47.39	47.71	47.56	47.86	2.6*	2.1;3.1	2013-2018
												-0.1*	-0.8;-0.3	2018-2023
Rondônia	49.29	54.76	52.08	49.74	51.37	52.47	52.53	51.84	53.03	51.41	51.04	0.1	-0.6;0.7	2013-2023
Roraima	37.50	39.14	51.77	50.44	51.21	52.08	51.15	52.98	52.68	51.02	49.96	20.7*	12.5;25.1	2013-2015
												0.3	-0.6;0.9	2015-2023
Tocantins	42.34	42.41	44.47	44.94	44.23	44.93	45.29	44.50	45.63	44.78	45.06	2.1*	1.3;3.8	2013-2016
												0.1	-0.3;0.3	2016-2023
Maranhão**	35.82	37.88	39.24	38.25	38.49	40.39	40.49	39.58	40.01	40.78	42.11	1.2*	0.6;1.8	2013-2023
Mato Grosso	45.80	47.34	50.69	50.84	52.92	53.99	54.38	52.53	52.89	52.64	52.46	4.9*	4.0;5.6	2013-2015
												2.6*	0.1;3.0	2015-2018
												-0.7*	-1.2;-0.5	2018-2023
Male	2013	2014	2015	2016	2017	2018	2019	2020	2021	2022	2023	APC	95%CI	Period
Legal Amazon	36.03	35.74	41.23	40.9	41.79	42.7	42.6	42.65	42.79	43.03	43.13	6.1*	4.7;8.2	2013-2016
												0.5*	0.1;0.8	2016-2023
Acre	33.33	34.12	36.95	41.97	38.94	40.75	40.38	40.54	39.23	42.43	41.02	7.2*	3.9;15.3	2013-2016
												0.4	-1.4;1.2	2016-2023
Amazonas	33.64	32.7	48.55	47.08	48.40	48.25	48.87	48.99	48.95	48.95	47.69	15.9*	12.0;24.5	2013-2016
												-0.2	-1.6;0.8	2016-2023
Amapá	44.44	66.67	44.58	41.27	42.99	44.05	42.2	47.93	47.58	45.95	48.46	-0.8	-4.1;2.6	2013-2023
Pará	36.78	37.86	41.79	41.45	42.43	42.95	43.17	42.76	43.05	43.06	43.16	6.7*	6.2;7.3	2013-2015
												1.4*	1.0;1.7	2015- 2018
												0.1	-0.3;0.2	2018-2023
Rondônia	40,00	43.48	45.67	42.16	43.78	45.11	44.66	44.85	46.14	44.38	44.19	0.7*	0.1;1.3	2013-2023
Roraima	41.18	0,00	47.39	44.44	43.85	46.56	45.91	48.03	47.59	45.82	43.23	0.5	-0.7;1.7	2013-2023
Tocantins	34.64	31.83	37.54	38.94	37.84	38.53	39.25	38.68	39.66	39.01	39.18	5.7*	3.1;11.2	2013-2016
												0.3	-1.1;1.1	2016-2023
Maranhão**	28.64	29.96	34.30	33.21	33.78	35.19	35.06	34.90	35.37	36.14	37.10	8.8*	5.5;11.2	2013-2015
												1.1*	0.7;1.4	2015-2023
Mato Grosso	39.67	39.65	43.63	44.64	46.42	47.59	47.28	46.40	46.76	46.9	47.00	4.9*	3.9;6.1	2013-2017
												-0.1	-0.7;0.5	2017-2023
Female	2013	2014	2015	2016	2017	2018	2019	2020	2021	2022	2023	APC	95%CI	Period
Legal Amazon	46.51	42.88	48.23	48.99	50.15	51.83	51.82	51.26	51.61	51.89	52.31	3.2*	2.3;5.1	2013-2018
												0.1	-1.8;0.9	2018-2023
Acre	41.15	41.08	43.94	47.33	48.65	48.35	49.26	48.28	49.85	51.91	52.08	5.4*	3.0;10.4	2013-2016
												1.4	-0.4;2.1	2016-2023
Amazonas	39.71	43.34	52.79	52.40	53.50	55.70	56.23	56.42	57.03	56.28	55.54	15.4*	11.4;17.9	2013-2015
												2.4*	1.4;3.6	2015-2019
												-0.2	-2.4;0.5	2019-2023
Amapá	47.62	58.36	52.21	50.38	49.24	53.63	50.99	56.58	54.70	54.24	55.40	0.7	-0.2;1.5	2013-2023
Pará	46.48	43.04	47.1	47.84	48.83	50.87	50.84	51.03	51.36	51.14	51.68	2.9*	2.1;4.9	2013 - 2018
												0.4	-1.9;1.2	2018 - 2023
Rondônia	55.13	55.77	56.86	55.77	56.98	57.76	58.08	57.86	58.66	57.17	56.75	0.7*	0.6;0.9	2013-2021
												-1.8*	-2.7;-0.6	2021-2023
Roraima	35.48	39.06	53.20	53.69	55.51	55.38	54.45	57.22	56.87	55.38	55.76	26.7*	16.0;33.3	2013-2015
												0.9	-0.3;1.7	2015-2023
Tocantins	47.43	45.97	48.94	49.48	48.99	49.77	49.82	59.55	50.80	49.97	50.42	1.1*	0.1;2.1	2013-2023
Maranhão**	39.31	38.17	41.66	41.06	41.08	43.27	43.69	42.99	43.43	44.18	45.79	1.5*	1.0;2.0	2013-2023
Mato Grosso	50.22	50.06	55.08	55.42	57.46	58.41	59.15	57.50	57.80	57.32	56.99	3.5*	2.9;4.3	2013-2018
												-0.8*	-1.9;-0.2	2018-2023

* Significantly different from zero at the alpha level = 0.05.

** Only municipalities in the Legal Amazon.
